# USP7 sustains hematopoietic stem cell homeostasis partially via PU.1 stabilization

**DOI:** 10.7150/ijbs.123712

**Published:** 2026-01-01

**Authors:** Huizhuang Shan, Youping Zhang, Xinhua Xiao, Wenxuan Wu, Yingying Wang, Chujiao Zhu, Wenhui Bai, Ziwei Zhang, Yuanhui Zhai, Li Yang, Yunzhao Wu, Hu Lei, Hanzhang Xu, Yanfei Luo, Liming Lu, Yingli Wu

**Affiliations:** 1 Institute for Translational Medicine on Cell Fate and Disease, Shanghai Ninth People's Hospital, Key Laboratory of Cell Differentiation and Apoptosis of National Ministry of Education, Department of Pathophysiology, Shanghai Jiao Tong University School of Medicine, Shanghai, 200025, China.; 2 Department of Clinical Laboratory Medicine, Guangdong Provincial People's Hospital (Guangdong Academy of Medical Sciences), Southern Medical University, Guangzhou, 510000, Guangdong, China.; 3 Hongqiao International Institute of Medicine, Shanghai Tongren Hospital, Shanghai Jiao Tong University School of Medicine, Shanghai, 200025, China.; 4 Shanghai Institute of Immunology, Shanghai Jiao Tong University School of Medicine, Shanghai, 200025, China.; 5 Department of Chemistry, University of Cambridge, Lensfield Road, CB2 1EW, Cambridge, UK.; 6 Department of Hematology and Oncology, Guangzhou Women and Children's Medical Center, Guangzhou Medical University, Guangzhou, 510000, Guangdong, China.

**Keywords:** hematopoietic stem cell, USP7, hematopoiesis, PU.1

## Abstract

Hematopoietic stem cell (HSC) self-renewal and lineage commitment are tightly controlled by post-translational mechanisms, but the contribution of deubiquitination to these processes remains unclear. Here, we define ubiquitin-specific protease 7 (USP7) as a critical regulator of HSC maintenance and hematopoietic homeostasis. Conditional *Usp7* deletion in murine HSCs triggered rapid stem cell depletion, multilineage cytopenias, and systemic hematopoietic failure. *Usp7*-deficient HSCs displayed defective quiescence, reduced competitive repopulation capacity, and aberrant lineage differentiation. Mechanistically, USP7 directly binds and deubiquitinates the transcription factor PU.1, shielding it from proteasomal degradation. Loss of USP7 destabilized PU.1, leading to suppressed expression of PU.1 target genes critical for HSC quiescence and lineage specification. In competitive transplants, USP7-null HSCs exhibited severely impaired self-renewal, marked by diminished engraftment and differentiation. Ectopic PU.1 expression partially restored HSC function, confirming the USP7-PU.1 axis as essential for HSC integrity. Our study identifies USP7 as a post-translational checkpoint in hematopoiesis and reveals a novel deubiquitination-dependent mechanism controlling stem cell fate. These findings highlight the USP7-PU.1 interaction as a potential therapeutic target for hematopoietic disorders.

## Introduction

Hematopoietic stem cells (HSCs) sustain lifelong blood production by balancing self-renewal with lineage commitment—a process whose dysregulation underlies bone marrow failure, leukemias, and myelodysplastic syndromes (MDS) 1, 2. While transcriptional networks governing HSC fate are well studied, post-translational mechanisms, particularly deubiquitination, remain poorly understood despite their potential to dynamically modulate protein stability and activity. Identifying these regulatory nodes is critical for targeting HSC-driven pathologies.

Ubiquitin-specific protease 7 (USP7), a deubiquitinating enzyme (DUB) linked to DNA repair 3 and oncogenesis 4, stabilizes hematopoietic transcription factors like GATA1 5, NOTCH1 6, and IKZF1 7, positioning it as a master regulator of differentiation. However, its role in HSC maintenance—distinct from lineage-specific functions—remains unexplored. This gap is striking, as USP7 substrates (e.g. p53 8, PTEN 9) regulate stem cell quiescence, which suggests broader roles in HSC integrity.

Here, we uncover USP7 as a guardian of HSC self-renewal. Using hematopoietic conditional *Usp7* knockout mice, we demonstrate that *Usp7* depletion triggers HSC exhaustion, multilineage cytopenias, and hematopoietic collapse. Mechanistically, beyond the known hematopoietic transcription factors regulated by USP7, we demonstrated that USP7 binds, deubiquitinates and stabilizes PU.1, a transcription factor essential for myeloid/lymphoid priming, and maintaining expression of quiescence-associated PU.1 targets. Restoring PU.1 partially rescues *Usp7*-deficient HSC defects, identifying PU.1 as an important downstream mediator of USP7. Our findings not only resolve a critical gap in HSC post-translational regulation but also propose USP7 as a therapeutic target for bone marrow failure and leukemias driven by PU.1 dysregulation.

## Methods

### Mice

C57BL/6 background* Usp7^fl/fl^* mice were achieved by inserting LoxP sites spanning the exon 3 of *Usp7* via homologous recombination utilizing a genetic construction developed by the Cyagen Biosciences Inc. To obtain hematopoietic-specific *Usp7* knockout mice, the *Usp7^fl/fl^* mice were subsequently crossed with transgenic lines expressing *Mx1-Cre* or *Scl-Cre.* To induce *Mx1-Cre* recombinase, poly (I:C) (Sigma, Louis, MO, USA) was administered at a dose of 10 mg/kg every other day by intraperitoneal injections for a total of 5 injections. The poly(I:C) was prepared at a concentration of 1 mg/ml in distilled water. For the *Scl-Cre* model, Tamoxifen (Sigma) was administered at a dose of 50 mg/kg every other day by intraperitoneal injection for 3 weeks. The Tamoxifen was dissolved in corn oil at 10 mg/ml. All mice used in the experiments were between 8 and 12 weeks of age. They were housed under standard conditions: a 12-hour light/dark cycle at room temperature, with free access to food and water. All animal procedures were conducted in compliance with and approved by the Guideline for Animal Care at Shanghai Jiao Tong University School of Medicine.

### Flow cytometry and sorting

Bone marrow (BM) and splenocytes were freshly harvested and passed through a 40-μm nylon mesh cell strainer (BD Biosciences). Peripheral blood (PB) samples were depleted of erythrocytes using ammonium-chloride-potassium (ACK) lysis buffer and subsequently washed and resuspended in phosphate-buffered saline (PBS). For immunophenotyping, cells were stained with appropriate fluorophore-conjugated antibodies according to the manufacturer's protocols. A complete list of antibodies employed in this study is provided in Supplementary Table 1. Cell cycle profiling was performed using a combination of Hoechst 33342 and Ki67-PE staining. Flow cytometry data acquisition was carried out on either a BD LSRII or BD Fortessa instrument (BD Biosciences), while cell sorting was conducted using a FACSAria II sorter (BD Biosciences). Acquired data were processed and analyzed with FlowJo software (version 10; Tree Star, Ashland, OR).

### Transplantation experiments

For competitive BM transplantation, under the *Mx1*-Cre condition, a mixture of 5 × 10^5^ donor BM cells (CD45.2^+^) from either control or *Mx1-Cre*; *Usp7^fl/fl^* mice and an equal number of competitor BM cells (CD45.1^+^) from C57BL/6 mice was transplanted into lethally irradiated (7.5 Gy X-ray) CD45.1^+^ C57BL/6 recipient mice. Post-transplantation, recipients were temporarily maintained on antibiotic water (0.16 g/mL Enrofloxacin HCL, MeilunBio^®^, Dalian, China) for 2 weeks. PB donor chimerism and lineage distribution were monitored by flow cytometry at 4-week intervals. Sixteen weeks later, the BM cells from recipient mice were subjected to flow-cytometric analysis to determine the donor frequency in hematopoietic stem/progenitor cells (HSPCs).

For secondary transplantation, sixteen weeks after the primary transplant, 1 × 10^6^ BM cells from the primary chimeric mice were transplanted into secondary lethally irradiated (7.5 Gy, X-ray) CD45.1^+^ C57BL/6 recipients. Donor chimerism in PB was similarly tracked every 4 weeks by flow cytometry.

For cell-autonomous transplantation, *Usp7* was deleted specifically in hematopoietic cells after transplanting 5 × 10^5^ control or *Mx1-Cre*; *Usp7^fl/fl^* mice BM donor cells (CD45.2^+^) were mixed with 5 × 10^5^ C57BL/6 BM competitor cells (CD45.1^+^) prior to injection into lethally irradiated (7.5 Gy, X-ray) CD45.1^+^ C57BL/6 recipient mice. *Usp7* deletion was induced specifically in the hematopoietic compartment six weeks later via intraperitoneal injection of poly(I:C) administered six times. PB and BM were collected for flow-cytometric analysis 4 weeks after the final poly(I:C) injection to evaluate donor chimerism in blood lineages and HSPCs.

For rescue experiments, lentivirus plasmid pLVX-IRES-GFP or pLVX-IRES-GFP-PU.1 was mixed with packaging plasmids (pMD2.G and psPAX2) at a ratio of 4:3:1 and transfected into HEK293T cells. Lentiviruses were utilized for the infection of control or* Scl-Cre*; *Usp7^fl/fl^* mice Lin^-^ BM donor cells (CD45.2^+^) by spinning infection in the presence of 4 μg/mL polybrene. Subsequently, 1 × 10^5^ infected donor cells were mixed with 2 × 10^5^ competitor BM cells (CD45.1^+^) and transplanted into lethally irradiated (7.5 Gy, X-ray) CD45.1^+^ C57BL/6 recipient mice. Donor cell engraftment in PB was monitored by flow cytometry every 4 weeks.

### Colony forming unit assays

BM cells isolated from either control or *Mx1-Cre; Usp7^fl/fl^* mice were seeded in MethoCult medium (M3434, Stemcell Technologies) at a density of 2 × 10^4^ cells per well in 6-well plates and cultured at 37 ºC. After 10 days of incubation, colony-forming units (CFUs) were enumerated under a Nikon microscope and classified according to lineage potential: burst-forming unit erythroid (BFU-E), colony-forming units of granulocyte-macrophage lineages (CFU-GM, CFU-G, and CFU-M), and multipotent colony-forming units encompassing granulocyte, erythroid, macrophage, and megakaryocyte lineages (CFU-GEMM).

### Homing

1-2×10^7^ BM cells from control and *Usp7^fl/fl^; Mx1-Cre* mice or control and *Usp7^fl/fl^; Scl-Cre* mice were labeled with 7.5 μM carboxyfluorescein succinimidyl ester (CFSE; Invitrogen) and transplanted into lethally irradiated recipient mice. Eighteen hours post-transplantation, the proportion of CFSE^+^ cells was quantified by flow cytometry. Measurements included total CFSE^+^ cells in BM and spleen, as well as CFSE^+^ LSK cells, CFSE^+^ LT-HSCs, CFSE^+^ ST-HSCs, CFSE^+^ MPPs in BM. The analysis utilized the following antibody panel for BM staining: anti-lineage-PerCP-Cy5.5, anti-Sca-1-PE-Cy7, anti-c-Kit-APC, anti-CD34-eFluor450 and anti-CD135-PE.

### Analysis of cell apoptosis

Cell apoptosis was assessed by staining with Annexin V-PE followed by DAPI, performed in accordance with the manufacturer's protocol. The antibodies used in these experiments are listed in Supplementary Table 1.

### Bromodeoxyuridine (BrdU) incorporation assay

To evaluate cellular proliferation, BrdU (Abcone) was administered intraperitoneally to mice at a dosage of 1 mg per 10 g body weight 16 h prior to sample collection. BM cells were then isolated and subjected to lineage depletion using a Biotin Mouse Lineage Depletion Cocktail (BD Biosciences), followed by surface staining for LT-HSC markers. For intracellular BrdU detection, cells were fixed and permeabilized using BD Cytofix/Cytoperm buffer at room temperature for 30 minutes, followed by incubation with BD Cytoperm Permeabilization Buffer Plus at 4 ºC for 10 minutes. After a subsequent re-fixation step, cells were treated with DNase (300 µg/mL, Sangon Biotech) at 37 ºC for 1 h to expose BrdU epitopes, and finally stained with FITC-conjugated anti-BrdU antibody (Biolegend) for 30 minutes at room temperature.

### Sequential 5-fluorouracil (5-FU) challenge assay

For sequential 5-FU (Sigma) challenge assay, one week after the last tamoxifen injection, the *Scl-Cre*; *Usp7^fl/fl^* and control mice received weekly intraperitoneal injections of 5-FU at a dose of 150 mg/kg until either their demise or the predetermined endpoint of the experiment.

### 5-FU killing assay

For 5-FU killing assay, the mice were intraperitoneally injected with a single injection of 5-FU at a dose of 150 mg/kg. PB samples were collected from the mice at different time points for complete blood counts analysis.

### Complete blood counts analysis

PB was collected from mice via orbital puncture into EDTA-coated capillary tubes. The PB was gently mixed by tapping the tube a few times. The samples were allowed to incubate at room temperature for 30 minutes prior to being analyzed with a HEMAVET 950 hematology analyzer.

### Cell culture and transfection

The human embryo kidney (HEK293T) and 32D cells were purchased from American Type Culture Collection (ATCC, Manassas, VA, USA). HEK293T cells were cultured in DMEM (BasalMedia, Shanghai, China) supplemented with 10% fetal bovine serum (FBS; Gemini, Woodland, CA, USA). 32D cells were maintained in RPMI-1640 (BasalMedia, Shanghai, China) containing 10% FBS and 10 ng/mL murine interleukin-3 (IL-3). All cells were incubated at 37 ºC in a humidified atmosphere of 5% CO_2_. Plasmid transfection of HEK293T cells was performed using polyethyleneimine (PEI; Sigma) according to the manufacturer's instructions.

### PCR and quantitative real-time PCR (qRT-PCR)

Genotyping and verification of Cre-mediated deletion efficiency were conducted via PCR with primer sets designed to distinguish the wild-type (WT) USP7 allele, the floxed exon 3, and the deleted exon 3 (primer sequences are provided in Supplementary Table 2). For the qRT-PCR assay, total RNA was extracted from cells using TRIzol reagent (Invitrogen, Carlsbad, CA, USA) and reverse transcribed using HiScript III RT SuperMix (+gDNA wiper; Vazyme Biotech Co., Ltd, Nanjing, China), following the manufacturer's protocol. The qRT-PCR assay was performed with ChamQ Universal SYBR qPCR Master Mix (Vazyme Biotech Co., Ltd) on an ABI 7900 Real-time PCR System. Gene expression levels were calculated using the 2^-ΔΔCt^ method and normalized to GAPDH as an internal control. All primer sequences used in this study are listed in Supplementary Table 3; primers were synthesized by Sangon Biotech.

### Plasmids and reagents

The Myc-tagged PU.1 and mutants or Flag-tagged PU.1 were generated by PCR amplification and subsequent cloning into the pLVX-puro backbone. Flag-tagged USP7^WT^, USP7^C223S^ (USP7 catalytic mutant) and GFP-tagged USP7 constructs (WT and mutants) were preserved for our laboratory as previously described 6. The HA-ubiquitin plasmid was obtained from Addgene. The pLVX-IRES-GFP and pLVX-IRES-GFP-PU.1 were generously provided by Prof. Shanhe Yu (Shanghai Jiao Tong University School of Medicine, Shanghai, China). The proteasome inhibitor MG132 and the protein synthesis inhibitor cycloheximide (CHX) were supplied by TargetMol Chemicals Inc. (Boston, MA, USA).

### Western blotting and co-immunoprecipitation (Co-IP) assays

Western blotting analysis was performed as previously described 6, 10. For standard protocols, cells were collected and lysed using 1× SDS lysis buffer. For low-cell-number experiments, cell processing included a PBS wash step and subsequent lysis in protease inhibitor-supplemented RIPA buffer, with 0.5-1 × 10^4^ cells used per western blot. Protein samples of equal quantity were resolved on an 8% polyacrylamide gel (Vazyme Biotech Co., Ltd) and subsequently electrotransferred onto a nitrocellulose membrane (GVS Filter Technology, Bologna, Italy). The membranes were probed overnight at 4℃ with the following primary antibodies: PU.1 (2266S, Cell Signaling Technology; 1:1000), USP7 (A300-033A, Bethyl Laboratories;1:1000), Flag (AE005, ABclonal; 1:1000), Myc (M047-3, MBL; 1:1000), HA (3724S, Cell Signaling Technology; 1:1000), GFP (sc-9996, Santa Cruz; 1:1000), GST (2624S, Cell Signaling Technology; 1:1000), β-Actin (66009-1-lg, ProteinTech; 1:10,000), and β-Tubulin (2146S, Cell Signaling Technology; 1:1000). Following primary antibody incubation, membranes were incubated with HRP-conjugated secondary antibodies (SA00001-1 or SA00001-2, ProteinTech; 1:10,000) and signals were detected using a ChemiDoc Imaging System (Bio-Rad, Hercules, CA, USA).

Co-IP was conducted as described previously 6. Briefly, cells were collected and lysed in RIPA lysis buffer (50mM Tris, pH 7.4; 150mM NaCl; 1% Nonidet P-40; 0.5% sodium deoxycholate; 0.1% SDS) supplemented with protease inhibitor cocktail (Beyotime) on a rotary shaker at 4 °C for 30 min. Following centrifugation at 12,000 rpm for 15 min at 4 °C, the resulting supernatants were incubated overnight at 4 °C with the indicated primary antibodies or control IgG on a rotary shaker. The next day, Protein A/G Plus agarose beads (Beyotime) were added and incubation continued for 3 h. After three washes with lysis buffer, the immunoprecipitated proteins were eluted in 2× SDS-PAGE loading buffer and subjected to western blotting analysis.

### GST Pull-down assay

Bacterial-expressed GST protein or GST-USP7 recombinant protein (SinoBiological Inc., Beijing, China) was immobilized onto anti-GST magnetic beads (MedChemExpress, Newark, NJ, USA). Separately, Flag-tagged PU.1 was expressed in HEK293T cells, purified with anti-Flag M2 affinity Gel (Sigma), and eluted with a 3× Flag peptide (Sigma). For the binding assay, the purified Flag-PU.1 underwent incubation in the presence of the GST or GST-USP7 bound beads in interaction buffer (10 mM Tris-HCl pH 8.0, 100 mM NaCl, 1 mM EDTA) at 4 °C for 6 h. After five washes, bound proteins were eluted by boiling in 1× SDS-PAGE loading buffer and analyzed by western blotting.

### Deubiquitination assay

In order to assess the deubiquitination of PU.1 protein, HEK293T cells were transfected with indicated plasmids for 48 h, then treated with MG132 (10 μM) for 4 h before lysis. Cell lysates were subjected to immunoprecipitation assays and western blotting.

*In vitro* deubiquitination assay were conducted as previously described 6. Briefly, HEK293T cells were transfected with indicated plasmids. Proteins were then purified under denaturing conditions (50 mM Tris-HCl pH 8.0; 50 mM NaCl; 10 mM DTT; 1 mM EDTA; 5% glycerol) via immunoprecipitation using an anti-Myc antibody coupled to Protein A/G Plus agarose beads. Subsequently, the ubiquitinated PU.1 proteins were incubated with purified recombinant USP7 protein in deubiquitination buffer for 2 h at 37 °C. This reaction was expired by boiling in 5× SDS-PAGE sample buffer for 10 min. Finally, the samples were analyzed by western blotting.

### Bioinformatic analysis

To comprehensively evaluate USP7 expression patterns across tissues and hematopoietic cell populations, we performed integrated bioinformatic analyses using multiple public databases. For murine *Usp7* expression profiling, we queried the BioGPS database (http://biogps.org/#goto=welcome; GeneAtlas MOE430, gcrma; Probeset: 1454949_at), with normalized data representing diverse tissues, organs, and cell subpopulations. Single-cell resolution analysis of mouse HSPCs was conducted using the Single Cell Expression Atlas database (https://www.ebi.ac.uk/gxa/sc/home), where expression values were visualized as dot plots with cell type-specific color coding. Human hematopoietic cell expression data were derived from the BloodSpot database (www.bloodspot.eu; Dataset: Normal human hematopoiesis, HemaExplorer), displaying microarray-based USP7 expression profiles across hematopoietic lineages as averaged values per cell type. Additionally, we analyzed single-cell RNA sequencing (scRNA-seq) data of human Lin-CD34/CD164 cells, with transcriptional dynamics visualized via SPRING plots, where nodes represent individual cells and trajectory edges denote early lineage commitment stages. All datasets were processed using default normalization pipelines from respective platforms.

### RNA-seq and data analysis

Four weeks after the last poly(I:C) injection, LT-HSCs (LSK, CD34^-^CD135^-^) were sorted from control and *Mx1-Cre; Usp7^fl/fl^* mice, with two biological replicates per group. RNAs were sequenced by Shanghai Majorbio Biotechnology Co., Ltd. The sequencing was performed on the Illumina Novaseq 6000 platform (Illumina, USA), utilizing the Illumina TruseqTM RNA sample prep Kit for library construction. The library preparation workflow comprised total RNA extraction, mRNA enrichment via Oligo(dT) beads, fragmentation, cDNA synthesis, and adaptor ligation, followed by sequencing on an Illumina platform to generate 2×150 bp paired-end reads. Clean-reads from each sample were aligned to the mouse reference genome GRCm38.p6 using HISAT2 software (version 2.1.0). Gene expression levels were quantified utilizing HTSeq software (version 0.9.1) with default parameters. Gene expression levels were estimated using FPKM, and differentially expressed genes were identified using the DESeq2 package in R, with a threshold of p-adjusted < 0.05 and Fold Change > 2.

### Statistical analysis

All graphs were produced using GraphPad Prism 8 software (GraphPad Software Inc., La Jolla, CA, USA). Survival outcomes following 5-FU treatment were compared using the log-rank test and presented as Kaplan-Meier curves. Between-group comparisons were performed using two-tailed paired Student's t tests (two-group comparison) or one-way ANOVA (multiple groups). Significance levels are indicated as follows: ^*^P < 0.05, ^**^P < 0.01, ^***^P < 0.001, or ^****^P < 0.0001.

## Results

### Deletion of *Usp7* affects normal hematopoiesis

**Figure 1:**
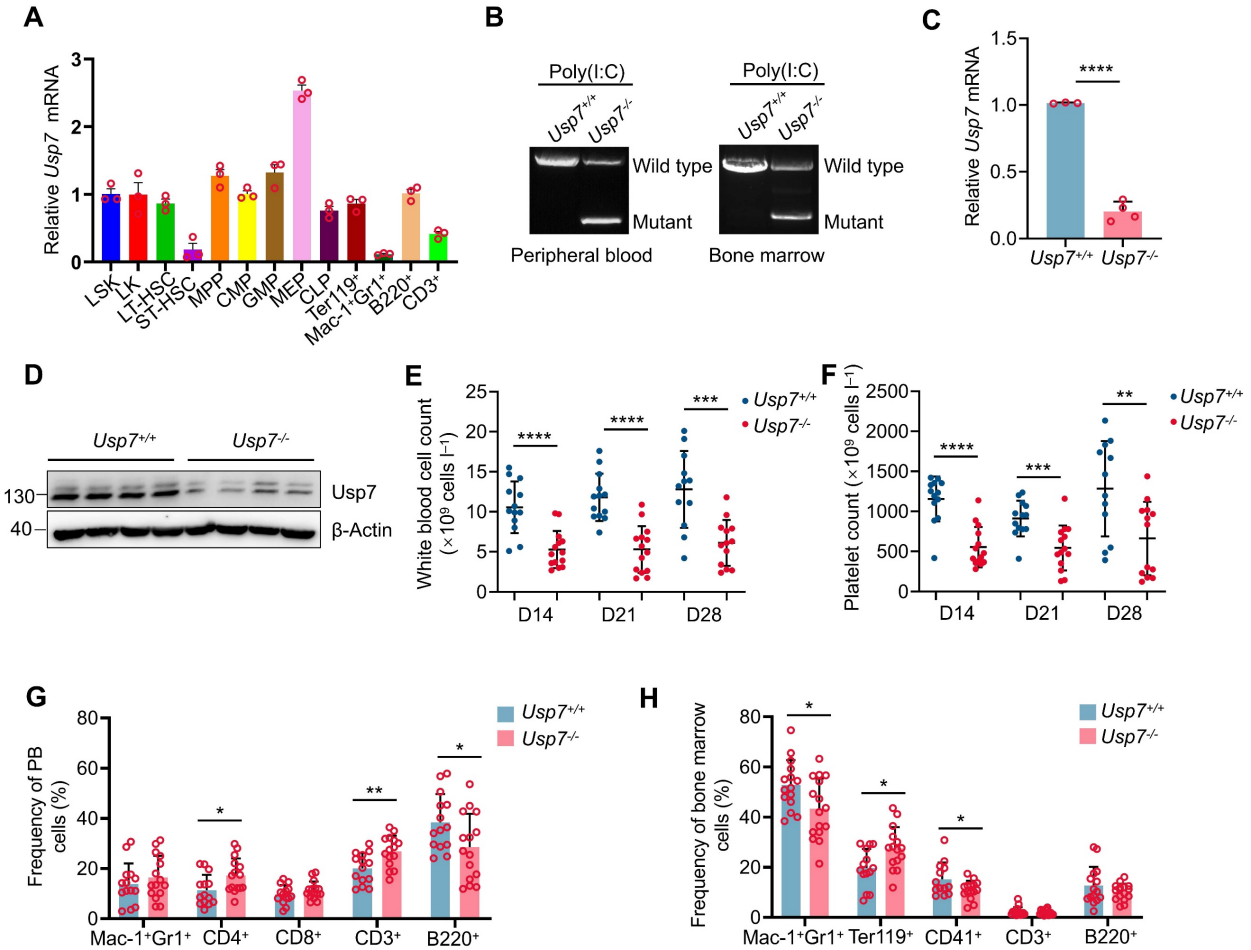
** Deletion of *Usp7* impairs normal hematopoiesis.** (**A**) Relative *Usp7* mRNA levels were measured by qRT-PCR in different hematopoietic populations. Cells were FACS-sorted from pooled 10 mice. (**B**) Representative genotyping results confirming *Usp7* deletion in peripheral blood (PB) cells (left) and bone marrow (BM) cells (right) from *Usp7^+/+^* and *Usp7^-/-^* mice 2 weeks after the last dose of poly (I:C). (**C**) *Usp7* deletion was examined by qRT-PCR in total BM cells from *Usp7^+/+^* and *Usp7^-/-^* mice (n = 3-4). (**D**) *Usp7* deletion was examined by western blotting in total BM cells from *Usp7^+/+^* and *Usp7^-/-^* mice (n = 4). (**E-F**) Whole blood counts of white blood cell (**E**) and platelet (**F**) counts from *Usp7^+/+^* and *Usp7^-/-^* mice at indicated times after the last dose of poly (I:C) (n = 13-14). (**G**) Frequency of differentiated cells in the PB from *Usp7^+/+^* and *Usp7^-/-^* mice at 4 weeks after the last poly (I:C) injection (n = 14-15). (**H**) Frequencies of mature cell populations in the BM from *Usp7^+/+^* and *Usp7^-/-^*mice at 4 weeks after the last poly (I:C) injection (n = 13-16). All data are presented as mean ± SD from at least 2 independent experiments. ^*^P < 0.05; ^**^P < 0.01; ^***^P < 0.001; ^****^P < 0.0001, as determined by unpaired two-tailed Student's t test.

An increasing body of evidence suggests that USP7 plays a significant role in embryogenesis and tumorigenesis; nevertheless, its expression and function within the hematopoietic system are not well understood, especially in HSCs. We initially queried the BioGPS public gene information database and found that *Usp7* was expressed across various tissues, organs, and hematopoietic cell subsets in mice, with relatively highest expression observed in the BM and megakaryocyte-erythroid progenitor (MEP) cells (Supplementary Fig. 1A). We further conducted single-cell expression analysis of USP7 using the Single Cell Expression Atlas database. The results revealed that *Usp7* was relatively highly expressed at the single-cell level in mouse BM, with similar expression levels in individual LT-HSCs and early lineage-committed progenitor cells (Supplementary Fig. 1B). Subsequently, we utilized the BloodSpot public gene expression database to analyze USP7 expression in various cell subsets of the normal human hematopoietic system. The findings demonstrated that USP7 was relatively highly expressed in HSCs (Supplementary Fig. 1C). Importantly, the expression pattern of USP7 in mice was conserved in humans, with USP7 expressed in human CD34^+^ HSCs and early lineage-committed progenitor cells at the single-cell level (Supplementary Fig. 1D). To corroborate the database findings, we quantified *Usp7* mRNA levels across distinct hematopoietic populations in C57BL/6 mouse BM. Notably, expression was elevated in HSPCs and peaked specifically within the MEP subset (Fig. 1A). These data suggest that USP7 may play an important role in hematopoiesis.

To test whether USP7 regulates hematopoiesis* in vivo*, we created *Usp7^fl/fl^* mice and bred them with *Mx1-Cre* transgenic mice, which induces *Usp7* deletion primarily within hematopoietic cells, yielding *Mx1-Cre*; *Usp7^fl/fl^* mice (Supplementary Fig. 1E-F). A highly efficient deletion of *Usp7* was achieved, as evidenced by genotyping, a dramatic reduction in mRNA expression (>80%) via qRT-PCR, and a corresponding drastic reduction in USP7 protein levels via western blotting 2 weeks following the final poly(I:C) injection (Fig. 1B-D). Hereafter, poly (I:C)-treated *Mx1-Cre^-^*; *Usp7^fl/fl^* and* Mx1-Cre*^+^;* Usp7^fl/fl^* were designated as *Usp7*^+/+^ and *Usp7^-/-^* mice, respectively. Considering that the *Mx1* promoter-driving Cre is expressed in both hematopoietic cells and stromal cells 11, we also crossed *Usp7^fl/fl^* mice with a tamoxifen-induced HSPCs specific *Scl-Cre* strain 12 to get *Scl-Cre; Usp7^fl/fl^* mice (Supplementary Fig. 1E,G), and the deletion of *Usp7* in *Scl-Cre; Usp7^fl/fl^* mice was similarly verified at 1 week after the last dose of tamoxifen (Supplementary Fig. 1H-I). Of great interest, complete blood count analyses demonstrated a marked reduction in white blood cell counts and platelet counts in *Usp7^-/-^* mice compared with *Usp7*^+/+^ mice, a phenomenon that persisted for 2 to 4 weeks following the final poly(I:C) injection (Fig. 1E-F), while red blood cell count, hemoglobin, and hematocrit remain unchanged (Supplementary Fig. 2A-C). Comparable results were also observed in *Scl-Cre; Usp7^fl/fl^* mouse model (Supplementary Fig. 2D-G). Recent studies have suggested that platelets can be produced directly from HSCs 13. This platelet phenotype indicates that USP7 may play a regulatory role in HSCs. Furthermore, we also found that *Usp7* deficiency led to a notable reduction in the frequencies of B220^+^ B cells and an elevation in the frequencies of CD3^+^ T cells in PB (Fig. 1G). Importantly, a significant decrease was observed in the BM frequencies of both Mac-1^+^Gr-1^+^ myeloid cells and CD41^+^ megakaryocytes, while the frequencies of erythroid cells were markedly increased (Fig. 1H). However, *Usp7* depletion did not trigger significant alterations in the frequencies of differentiated lineages except a reduction in B cells in spleens (Supplementary Fig. 4A). Overall, these findings demonstrate that *Usp7* deletion exerts a pronounced effect on normal hematopoiesis and the function of USP7 in hematopoiesis is lineage-specific and context dependent.

### *Usp7* deficiency impairs HSCs pool and promotes quiescent HSCs into cycling

**Figure 2:**
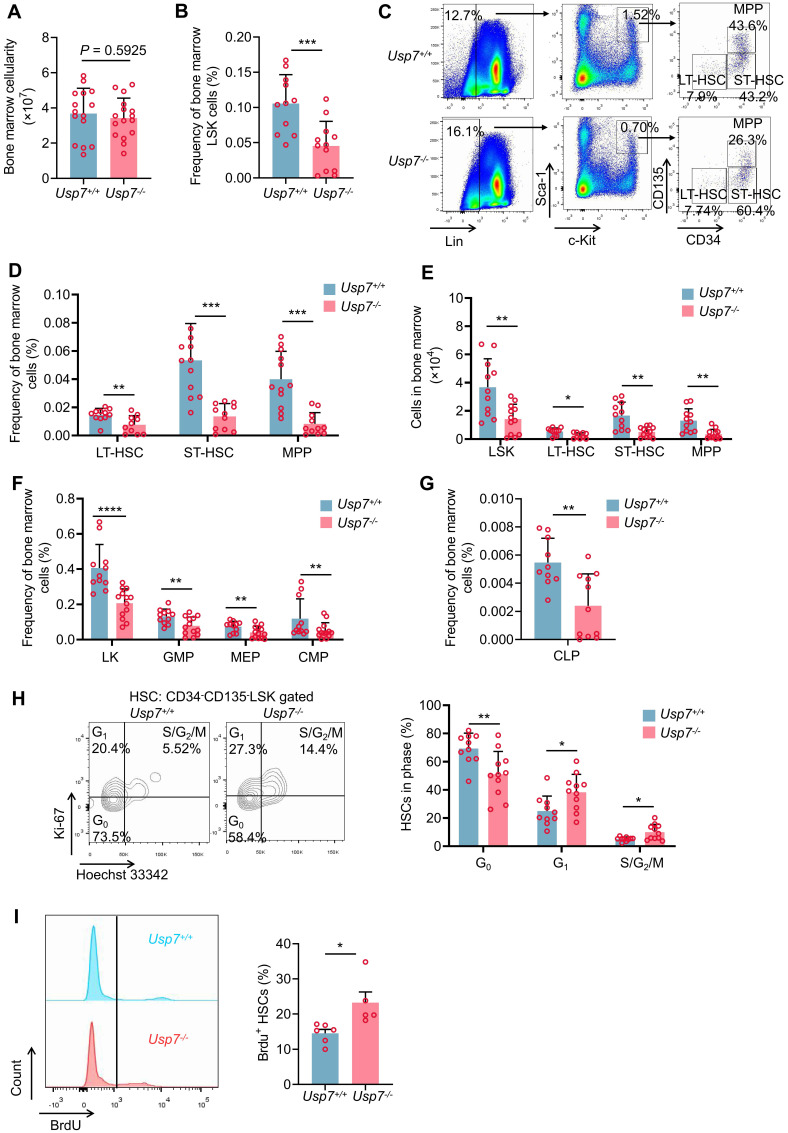
***Usp7* deficiency impairs HSCs pool and promotes quiescent HSCs into cycling.** (**A**) Total numbers of BM cells were counted in *Usp7^+/+^* and *Usp7^-/-^* mice at 4 weeks after the last poly (I:C) injection (n = 15-16) . (**B**) Frequency of LSK (Lin^-^Sca-1^+^c-Kit^+^) cells in BM from *Usp7^+/+^* and *Usp7^-/-^* mice at 4 weeks after the last poly (I:C) injection (n = 12). (**C-D**) Representative FACS plots (**C**) and frequencies (**D**) of LT-HSCs (LSK, CD34^-^CD135^-^), ST-HSCs (LSK, CD34^+^CD135^-^), and MPPs (LSK, CD34^+^CD135^+^) in BM from *Usp7^+/+^* and *Usp7^-/-^* mice at 4 weeks after the last poly (I:C) injection (n = 12). (**E**) Absolute numbers of LSK cells, LT-HSCs, ST-HSCs and MPPs in BM from *Usp7^+/+^* and *Usp7^-/-^* mice at 4 weeks after the last poly (I:C) injection (n = 12) . (**F**) Frequencies of LK (Lin^-^Sca-1^-^c-Kit^+^) cells, CMPs (LK, CD34^+^CD16/32^-^), GMPs (LK, CD34^+^CD16/32^+^), and MEPs (LK, CD34^-^CD16/32^-^) in BM from *Usp7^+/+^* and *Usp7^-/-^* mice at 4 weeks after the last poly (I:C) injection (n = 12-13) . (**G**) Frequency of CLPs (Lin^-^Sca-1^low^c-Kit^low^CD127^+^CD135^+^) in BM from *Usp7^+/+^* and *Usp7^-/-^* mice at 4 weeks after the last poly (I:C) injection (n = 11-13). (**H**) Cell cycle analysis of LT-HSCs in BM from *Usp7^+/+^*and *Usp7^-/-^* mice at 4 weeks after the last poly (I:C) injection (n = 10-11). Representative FACS plots (left) and percentages of different cell cycle phases (right) were calculated. (**I**) Proliferation assay of the LT-HSCs in *Usp7^+/+^* and *Usp7^-/-^* mice at 4 weeks after the last poly (I:C) injection (n = 5-6) . Representative FACS plots for BrdU staining (left) and percentages of BrdU^+^ cells (right) were showed. All data are presented as mean ± SD from at least 2 independent experiments. ^*^ *P* < 0.05; ^**^P < 0.01; ^***^P < 0.001; ^****^P < 0.0001, as determined by unpaired two-tailed Student's t test.

Having shown a defect in hematopoiesis in *Usp7* knockout mice and given that various blood cells are derived from the differentiation of HSPCs, we opted to assess the possible influences of *Usp7* deletion on HSPCs. The overall BM cellularity remained largely unchanged following *Usp7* deletion (Fig. 2A). However, both the frequencies and absolute numbers of LSK (Lin^-^Sca-1^+^c-Kit^+^) cells—encompassing LT-HSCs, ST-HSCs, and MPPs—were significantly reduced in *Usp7^-/-^* mice relative to *Usp7*^+/+^ mice at 4 weeks post the last poly (I:C) injection (Fig. 2B-E). Moreover, *Usp7* deletion also led to the reduced frequencies of LK (Lin^-^Sca-1^-^c-Kit^+^) progenitor populations, including CMPs, GMPs, MEPs, and CLPs (Fig. 2F-G). Consistently, we observed similar results in *Scl-Cre; Usp7^fl/fl^* mouse model (Supplementary Fig. 3A-D). Afterward, we also examined the cellularity and the frequencies of HSPCs in the spleens. As shown in Supplementary Fig. 4B-D, although the total spleen cellularity of *Usp7^-/-^* mice was significantly lower compared to the controls, the frequencies of LSK and LT-HSCs remain unchanged. Meanwhile, the total colony numbers of *Usp7^-/-^* BM cells, particularly megakaryocyte progenitor cells (CFU-M colonies), were significantly decreased (Supplementary Fig. 5A-B). In contrast, colonies derived from other primitive myeloid progenitor cells (CFU-GEMM colonies) or differentiated myeloid lineages (BFU-E, CFU-G, CFU-GM colonies) were unchanged compared to the control counterparts (Supplementary Fig. 5A-B).

To elucidate the cause of the diminished HSC frequency in *Usp7^-/-^* HSCs, we analyzed the cell cycle status of HSCs through Hoechst 33342 and Ki67 staining and found that approximately 58% of LT-HSCs were in G_0_ in *Usp7^-/-^* mice compared with 73% in *Usp7^+/+^* mice (Fig. 2H). *In vivo* BrdU incorporation labeling also showed the relatively higher proliferation rate of *Usp7^-/-^* LT-HSCs (Fig. 2I). These results suggest that *Usp7^-/-^* HSCs exhibit reduced quiescence and an increased propensity for division and proliferation. Conversely, no significant difference in apoptosis was detected between *Usp7^-/-^* and *Usp7^+/+^* LT-HSCs (Supplementary Fig. 5C). Collectively, our data suggest that *Usp7* deletion in HSCs promotes quiescent HSCs into cycling.

### *Usp7* deletion compromises the long-term repopulation capacity of HSCs

**Figure 3:**
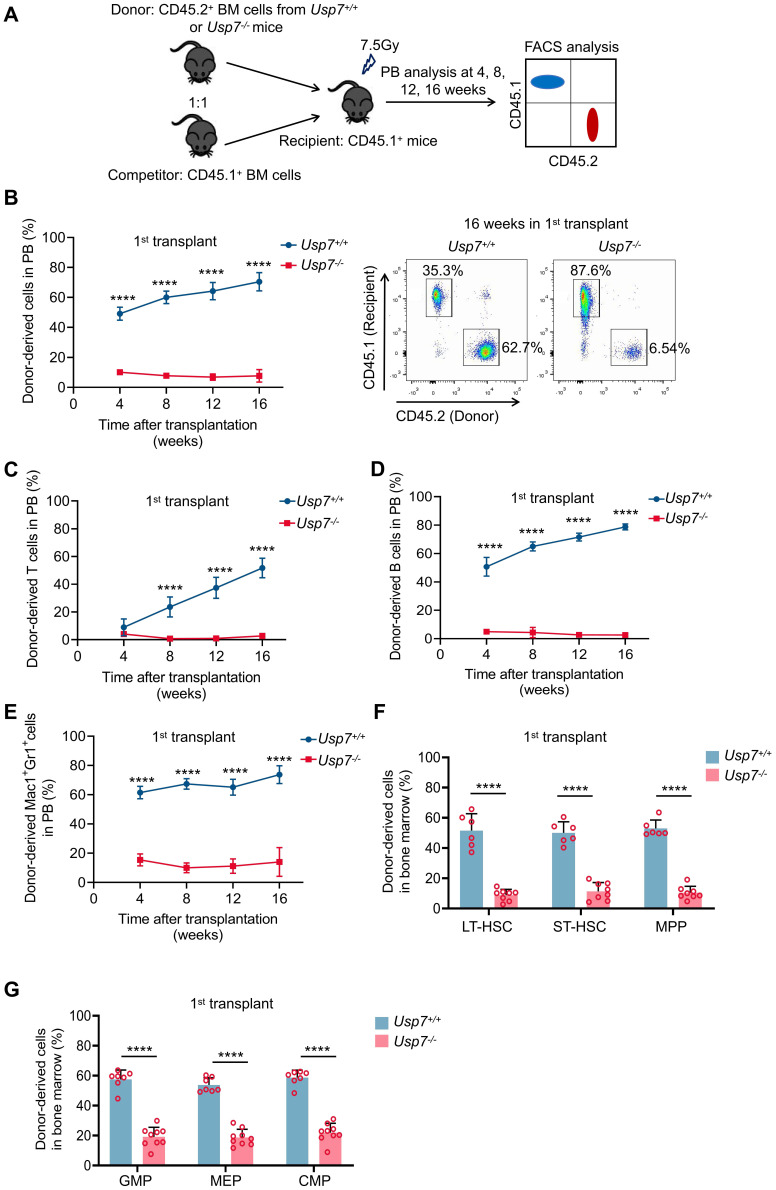
** Loss of *Usp7* disrupts the long-term repopulation capacity of HSCs.** (**A**) Schematic of the competitive transplantation assay. 5 × 10^5^ donor whole BM cells from *Usp7^+/+^* or *Usp7^-/-^* mice with 5 × 10^5^ competitor cells were transplanted into CD45.1 recipient mice. The multi-lineage chimaera levels in PB were assessed at different time points up to 16 weeks after transplantation. (**B**) Percentage of donor-derived cells (left) in PB were measured at different time points after HSC transplantation from recipient mice (n = 7-9). Representative FACS plots for the proportion of donor cells in PB of recipient mice at 16 weeks after transplantation. (**C-E**) Percentage of donor-derived T cells (**C**), B cells (**D**), and Mac-1^+^Gr-1^+^ myeloid cells (**E**) in PB were measured at different time points after HSC transplantation from recipient mice (n = 7-9). (**F**) Frequencies of donor-derived LT-HSCs, ST-HSCs and MPPs in BM were analyzed at 16 weeks after transplantation (n = 6-8). (**G**) Frequencies of donor-derived GMPs, MEPs and CMPs in BM were analyzed at 16 weeks after transplantation (n = 6-8). All data are presented as mean ± SD from at least 2 independent experiments. ^*^ *P* < 0.05; ^**^P < 0.01; ^***^P < 0.001; ^****^P < 0.0001, as determined by unpaired two-tailed Student's t test.

To further test the impact of *Usp7* deletion on HSC long-term repopulation capacity, we competitively transplanted BM cells from *Usp7^+/+^* or *Usp7^-/-^* mice (CD45.2^+^) combined with competitor cells (CD45.1^+^) into lethally irradiated recipient mice (CD45.1^+^). Donor-derived cell repopulation and lineage distribution in PB were then monitored at 4, 8, 12, and 16 weeks post-transplantation (Fig. 3A). Our results showed that repopulation by *Usp7^-/-^* BM cells was significantly reduced relative to *Usp7^+/+^* cells at indicated times after transplant (Fig. 3B). Of note, a profound reduction in donor contribution to T cells (Fig. 3C), B cells (Fig. 3D), and myeloid cells (Fig. 3E) was observed in recipients of *Usp7^-/-^* BM cells, implying that* Usp7* deletion affects HSCs differentiation. *Usp7^-/-^* HSPCs, comprising LT-HSCs, ST-HSCs, MPPs, GMPs, MEPs and CMPs, also displayed a substantial decrease at 16 weeks post-transplantation (Fig. 3F-G). Following transplantation into secondary recipients, the results exhibited a comparable repopulation trend (Supplementary Fig. 6A-D), indicating that *Usp7* deletion results in impaired self-renewal capacity of HSCs.

To examine whether the decreased repopulation capacity was caused by homing defects in *Usp7^-/-^* HSCs, we first performed homing assays using CFSE-labeled total BM cells. No significant differences were observed between the *Usp7^+/+^* and *Usp7^-/-^* donors in the BM at 18 h after transplant; however, a marked decrease in *Usp7^-/-^* donors was noted within the spleens (Supplementary Fig. 6E), suggesting a tissue-specific role for USP7. Since the homing behavior of rare HSCs might be obscured in bulk population analyses, we employed an *Scl-Cre; Usp7^fl/fl^* mouse model to specifically assess the homing efficiency of defined HSCs and progenitor populations. Consistent with the total BM results, the frequencies of homed CFSE^+^ cells within the LSK compartment, as well as LT-HSCs, ST-HSCs, and MPPs, were comparable between control and *Scl-Cre*; *Usp7^fl/fl^* mice in the BM (Supplementary Fig. 6F-G). This combined evidence demonstrates that USP7 deficiency does not impair the intrinsic homing capacity of HSCs to the BM. In summary, these data indicate that the reduced long-term repopulation potential of *Usp7*-deficient HSCs is not due to impaired homing.

### *Usp7* is required for cell-autonomous HSC function

**Figure 4:**
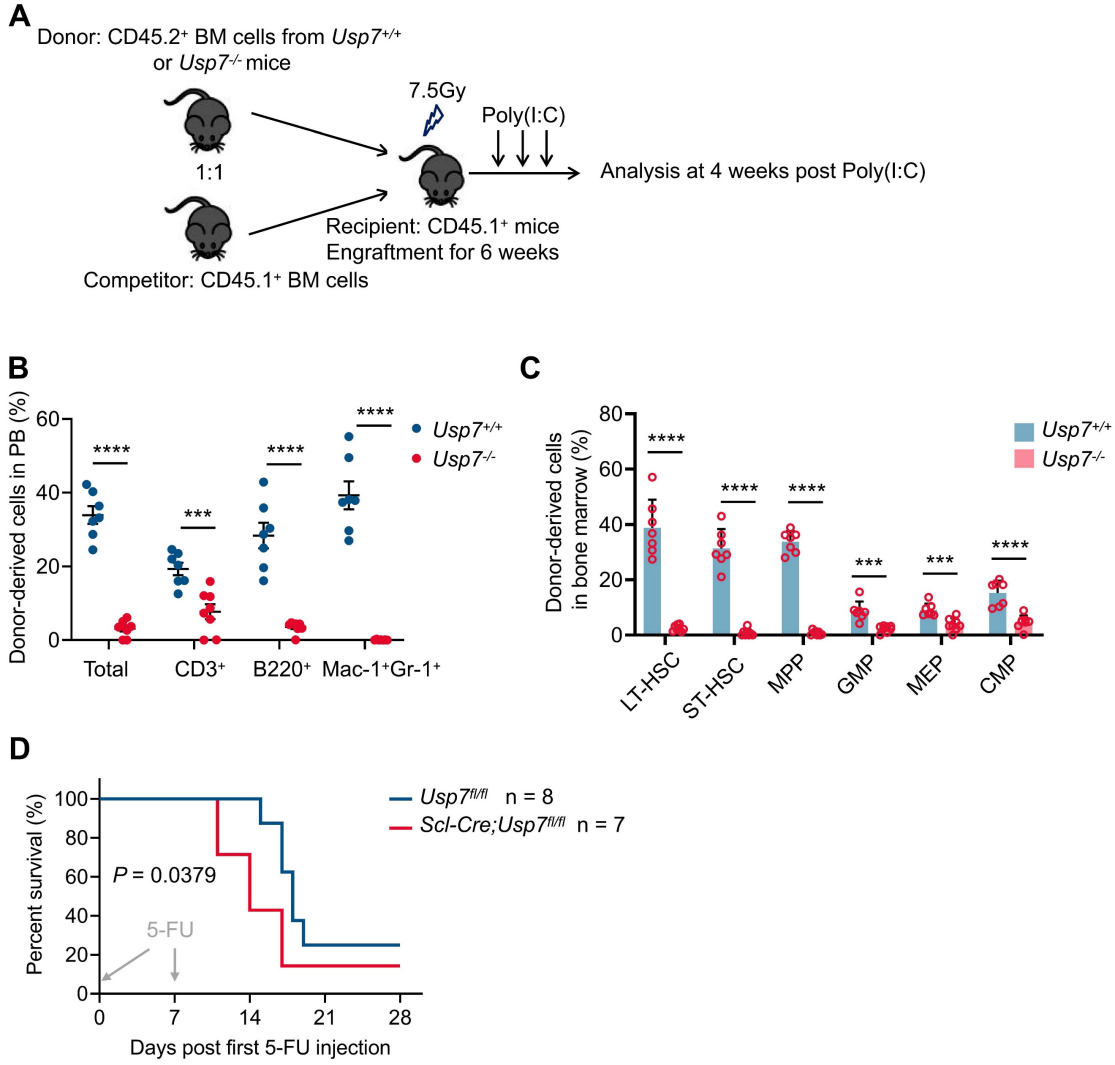
***Usp7* regulates HSCs self-renewal and differentiation in a cell-autonomous manner.** (**A**) Schematic of the experimental procedure in (**B**) and (**C**). CD45.1 recipient mice were transplanted with *Usp7^+/+^* or *Usp7^-/-^* BM cells (pre-poly (I:C)). At 6 weeks post-transplantation, CD45.1 recipient mice were then injected with poly (I:C) to deplete *Usp7*. (**B**) Percentage of donor-derived total cells, T cells, B cells, and Mac-1^+^Gr-1^+^ myeloid cells in PB from recipient mice were measured at 4 weeks post-poly (I:C) (n = 7-8). (**C**) Frequencies of donor-derived LT-HSCs, ST-HSCs, MPPs, GMPs, MEPs and CMPs in BM from recipient mice were analyzed at 4 weeks post-poly (I:C) (n = 7-8). (**D**) For sequential 5-FU challenge assay, one week after the last tamoxifen injection, the control or *Scl-Cre*;*Usp7^fl/fl^* mice were intraperitoneally administered 5-FU (150 mg/kg) weekly for 2 times (arrows), and the survival rates were analyzed (n = 7-8). All data are presented as mean ± SD from at least 2 independent experiments.

To determine whether *Usp7* is cell-autonomously necessary for HSCs function, we conducted competitive BM transplantations. BM cells from *Usp7^+/+^* or *Usp7^-/-^* mice (CD45.2^+^) were mixed with competitor cells (CD45.1^+^) and transplanted into lethally irradiated recipient mice (CD45.1^+^), without initially inducing *Usp7* deletion. Six weeks post-transplantation, *Usp7* deletion was triggered in recipients by poly(I:C) administration. Four weeks after the final poly(I:C) dose, chimerism analysis was performed on PB and BM cells from the recipient mice (Fig. 4A). We found a dramatic reduction in donor engraftment in PB and BM by* Usp7-*deficient BM cells (Fig. 4B-C). Further analysis of donor contribution to PB lineages revealed a significant decrease in T cells and an almost complete loss of B cells and myeloid cells. (Fig. 4B).* Usp7-*deficient HSPCs, comprising LT-HSCs, ST-HSCs, MPPs, GMPs, MEPs and CMPs, also exhibited a dramatic reduction (Fig. 4C).

We subsequently investigated the impact of USP7 ablation on normal hematopoiesis under conditions of hematopoietic stress. Sequential 5-FU challenge assay revealed that *Scl-Cre*; *Usp7^fl/fl^* mice died dramatically earlier than *Usp7^fl/fl^* mice (Fig. 4D). In line, 5-FU killing assay indicated that at 2 weeks after a single injection of 5-FU, the recovery rate of neutrophils in *Scl-Cre*; *Usp7^fl/fl^* mice was significantly slower compared to the control mice (Supplementary Fig. 7A-C). These findings indicate that *Usp7* is essential for cell-autonomous HSC functionality and the consequences of *Usp7* ablation on normal hematopoiesis become more pronounced under conditions of hematopoietic stress.

### Identification of *Usp7* targets in HSCs

**Figure 5:**
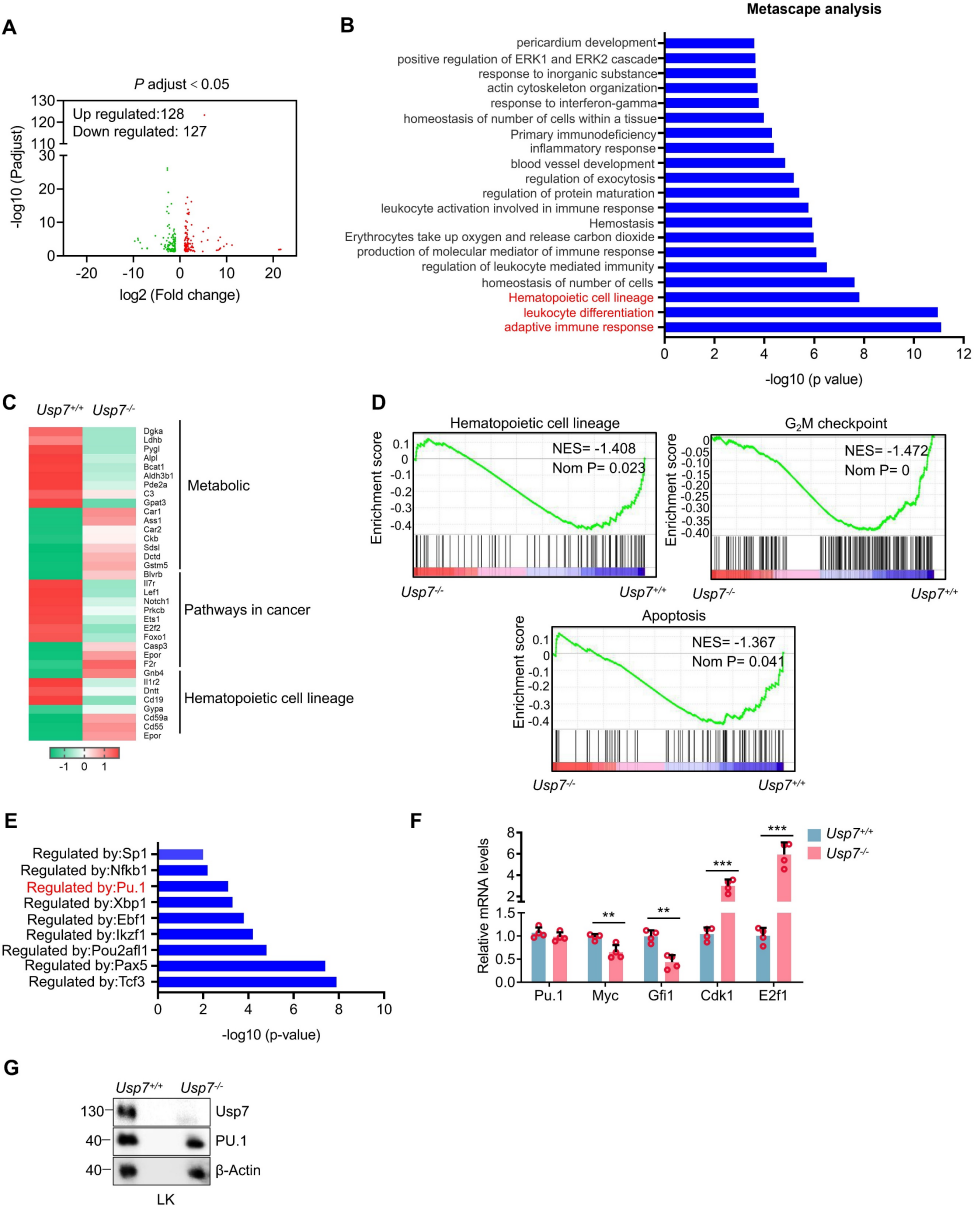
** Identification of *Usp7* regulation targets in HSCs.** BM LT-HSCs were isolated from *Usp7^+/+^* and *Usp7^-/-^* mice 4 weeks at after the last poly (I:C) injection and analyzed by RNA-Seq. (**A**) Volcano plot analysis of all transcripts in *Usp7^+/+^* vs *Usp7^-/-^* HSCs. (**B**) Metascape analysis of the regulating genes enriched in the top 20 signaling pathways. Candidate changes are highlighted in red. (**C**) Heatmaps of the differentially expressed genes after *Usp7* deletion related to metabolic, pathways in cancer, and hematopoietic cell lineage. Heatmap scale represents z score. (**D**) Gene set enrichment analysis (GSEA) showing that genes were downregulated in *Usp7^-/-^* HSCs are significantly correlated with genes associated with hematopoietic cell lineage, G_2_/M checkpoint, and apoptosis. (**E**) Metascape analyses of the regulating genes enriched in the top 8 transcription factor targets. PU.1 is highlighted in red. (**F**) qRT-PCR analysis of the indicated genes in *Usp7^+/+^* or *Usp7^-/-^* HSCs (n = 4). (**G**) Western blotting of USP7 and PU.1 in sorted LK cells from *Usp7^+/+^* or *Usp7^-/-^* mice.

To identify potential targets through which USP7 regulates HSCs function, we performed RNA-seq analysis on sorted BM LT-HSCs from *Usp7^+/+^* and *Usp7^-/-^* mice. The analysis revealed 128 upregulated and 127 downregulated genes in *Usp7*-deficient LT-HSCs (Fig. 5A). Metascape analysis showed that the top 3 most significantly enriched pathways are linked to adaptive immune response, leukocyte differentiation and hematopoietic cell lineage (Fig. 5B). The heatmaps results created from KEGG database focusing on the top 3 most significantly enriched pathways revealed that genes associated with metabolic pathways, pathways in cancer, and hematopoietic cell lineage were up-regulated or down-regulated to varying degree (Fig. 5C). Additionally, GSEA results revealed a significant down-regulation trend in gene sets related to hematopoietic cell lineage, the G_2_/M checkpoint of the cell cycle, and apoptosis in the HSCs of *Usp7^-/-^* mice (Fig. 5D). To experimentally validate this finding from the hematopoietic cell lineage pathway, we performed qRT-PCR on sorted HSCs and confirmed the significant downregulation of key pathway genes, including *Flt3*, *Gata1*, and *c-Kit* (Supplementary Fig. 8). As a deubiquitinase, USP7 has been shown to be involved in regulating the stability and activity of various transcription factors 14-16. Importantly, we conducted transcription factor regulatory analysis on the differentially expressed genes from the RNA-Seq results and found that the target genes of several transcription factors exhibited significant changes in *Usp7*-deficient HSCs (Fig. 5E). We noticed that PU.1 (also known as SPI1), which is a determinant factor for myeloid differentiation and plays a crucial regulatory role in hematopoietic development 17, 18, was consistently identified as one of the most abundant transcription factors regulated by USP7. As shown in Fig. 5F, compared to the *Usp7^+/+^* HSCs, the mRNA level of PU.1 in *Usp7^-/-^* HSCs remained unchanged, but its target genes exhibited significant alterations, characterized by a marked downregulation of *Myc* and *Gfi1*, as well as a significant upregulation of *Cdk1* and *E2f1*. However, despite the unaltered mRNA level, the protein abundance of PU.1 was substantially decreased in sorted *Usp7*^-/-^ LK cells (Fig. 5G), indicating a post-transcriptional mechanism of regulation by USP7. Therefore, these data demonstrate that PU.1 may contribute to USP7 knockout caused alteration in normal hematopoiesis.

### USP7 interacts and deubiquitinates PU.1

**Figure 6:**
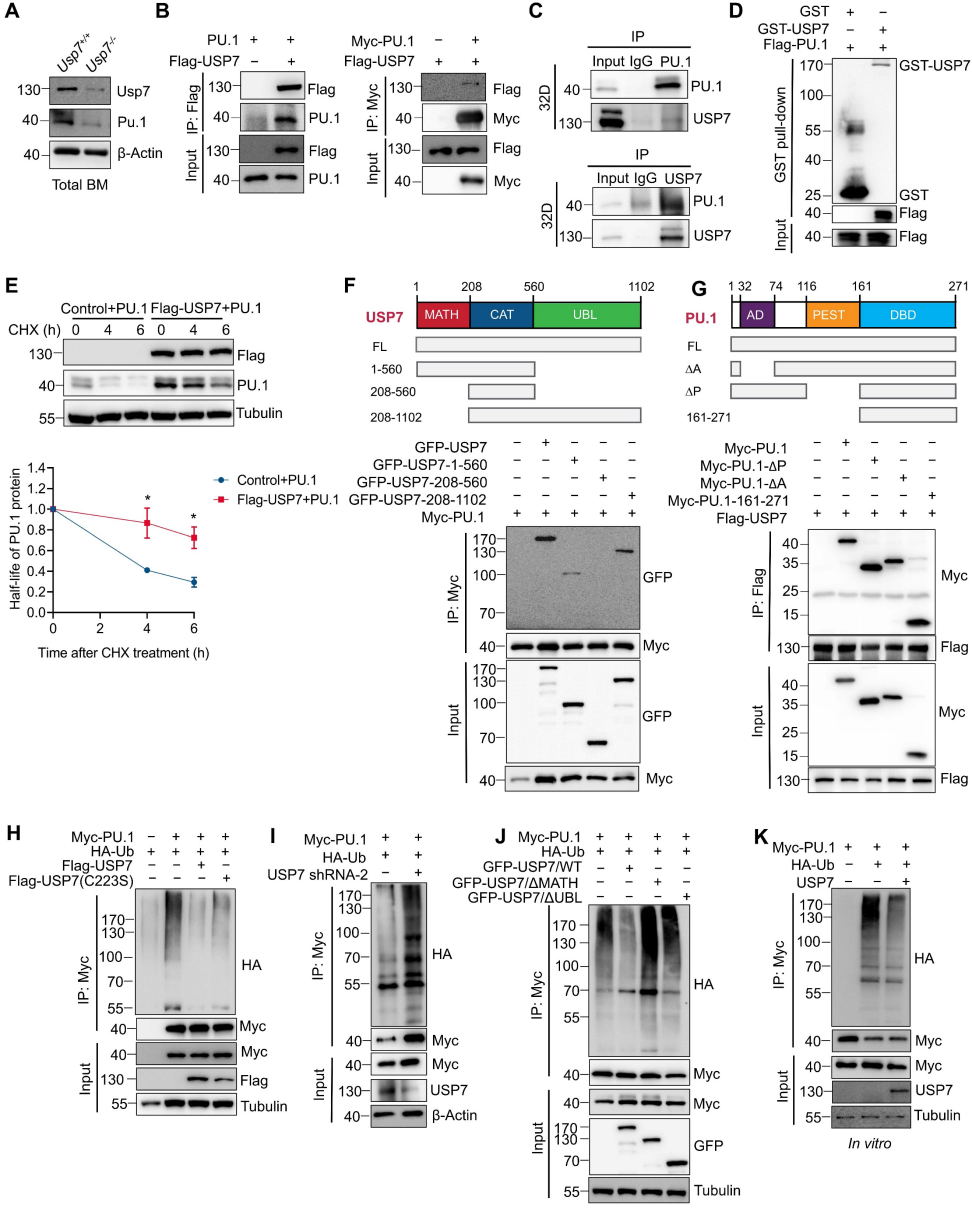
** USP7 directly interacts with and deubiquitinates PU.1.** (**A**) Analysis of USP7 and PU.1 protein expression by western blotting in total BM cells from *Usp7^+/+^* or *Usp7^-/-^* mice. (**B**) USP7 and PU.1 interaction in HEK293T cells transfecting with the indicated plasmids. (**C**) Lysates from 32D cells were immunoprecipitated using either an anti-PU.1 (upper panel) or an anti-USP7 antibody (lower panel), and followed by western blotting with antibodies against the indicated proteins. (**D**) The direct interaction between USP7 and PU.1 was detected using a GST pull-down assay, and the indicated proteins were examined by western blotting. (**E**) PU.1 protein stability in HEK293T cells overexpressing PU.1 and Flag-USP7 (upper panel). The intensity of the PU.1 bands was quantified by Image J software (lower panel). (**F**) Structure schematic diagram of full-length and truncated segments of USP7 (upper panel). Lysates from HEK293T cells co-transfected with Myc-PU.1 and either full-length GFP-USP7 or its truncated mutants were subjected to Co-IP with anti-Myc antibodies (lower panel). (**G**) Structure schematic diagram of full-length and truncated segments of PU.1 (upper panel). Lysates from HEK293T cells co-transfected with Flag-USP7 and either full-length Myc-PU.1 or its truncated mutants were subjected to Co-IP with anti-Flag antibodies (lower panel). (**H**) HEK293T cells expressing the indicated plasmids and then subjected to Co-IP assays with anti-Myc antibody followed by western blotting with anti-HA antibody. (**I**) HEK293T cells stably expressing shRNA specifically against USP7 were co-transfected with the indicated plasmids. Co-IP was performed with anti-Myc antibody followed by western blotting with anti-HA antibody. (**J**) HEK293T cells were co-transfected with the indicated plasmids. Co-IP was performed with anti-Myc antibody followed by western blotting with anti-HA antibody. (**K**)* In vitro* deubiquitination assays. HEK293T cells were co-transfected with HA-Ub and Myc-PU.1. Ubiquitinated-PU.1 proteins were purified using an anti-Myc antibody and then incubated with purified USP7 protein at 37 °C for 2 h, followed by western blotting with anti-HA antibody.

Given that PU.1 is a critical determinant of HSC differentiation 19, 20 and considering the phenotypic parallels observed in both *Pu.1* and *Usp7* conditional knockout models (reduced quiescence and diminished repopulating capacity of HSCs), we examined whether PU.1 is regulated by USP7. The downregulation of PU.1 protein in *Usp7*-deficient LK cells (Fig. 5G) and *Usp7*-deficient total BM cells (Fig. 6A) supports this notion. Using a Co-IP assay, we investigated the interactions between USP7 and PU.1. The results showed that USP7 and PU.1 reciprocally co-immunoprecipitated (Fig. 6B). Furthermore, an interaction between endogenous USP7 and PU.1 was also detectable in 32D cells, a mouse myeloid progenitor cell line (Fig. 6C). As shown in Fig. 6D, an *in vitro* GST-pull down assay demonstrated that GST-USP7 but not GST alone, pulled down Flag-PU.1, supporting a direct interaction of PU.1 with USP7 protein. Moreover, USP7 overexpression in HEK293T cells substantially suppressed the CHX-induced degradation of PU.1 protein (Fig. 6E). These results indicate that USP7 directly interacts with PU.1.

To delineate the domains of USP7 and PU.1 necessary for their interaction, GFP-tagged full-length, TRAF homology (MATH) domain (amino acids 1-208), catalytic domain (amino acids 208-560), and ubiquitin-like (UBL) domains (amino acids 560-1102) of USP7 (Fig. 6F) were co**-**transfected with Myc-tagged PU.1 into HEK293T cells, followed by Co-IP with anti-Myc antibody. As depicted in Fig. 6F, PU.1 interacted with full-length USP7 and its MATH and UBL domains, but not with the catalytic domain, demonstrating that both the N-terminal MATH and C-terminal UBL domains are necessary for this interaction. Next, cells were co-transfected Myc-tagged full-length, ∆AD, ∆PEST, and DBD domain of PU.1 (Fig. 6G) with Flag-USP7, followed by Co-IP with anti-Flag antibody. The results demonstrate that the C-terminal DBD domain of PU.1 did not interact with USP7 (Fig. 6G).

Further, we sought to investigate whether USP7 acts as a deubiquitinase for PU.1. We first co-transfected HEK293T cells with plasmids expressing HA-ubiquitin and Myc-PU.1, together with either wild-type USP7 (USP7^WT^) or its catalytically-inactive mutant (USP7^C223S^). Co-expression of USP7^WT^, but not USP7^C223S^, substantially reduced the levels of ubiquitinated-PU.1 (Fig. 6H). Conversely, knockdown of USP7 using specific shRNAs in HEK293T cells increased PU.1 ubiquitination under the same experimental settings (Fig. 6I). We next examined whether the MATH and UBL domains of USP7 are required for its deubiquitinase activity toward PU.1. As shown in Fig. 6J, neither the MATH- nor the UBL-deleted USP7 mutants could remove ubiquitin chains from PU.1, indicating that both domains are essential for this function. To further validate USP7 as a direct DUB for PU.1, we performed an* in vitro* deubiquitination assay. Ubiquitinated-PU.1 proteins were purified from HEK293T cells and incubated with commercially available purified USP7 protein. As illustrated in Fig. 6K, the purified USP7 markedly reduced the ubiquitination of PU.1. Taken together, these results indicate that PU.1 is a bona fide substrate of USP7.

### USP7 regulates HSC function partially by modulating PU.1

**Figure 7:**
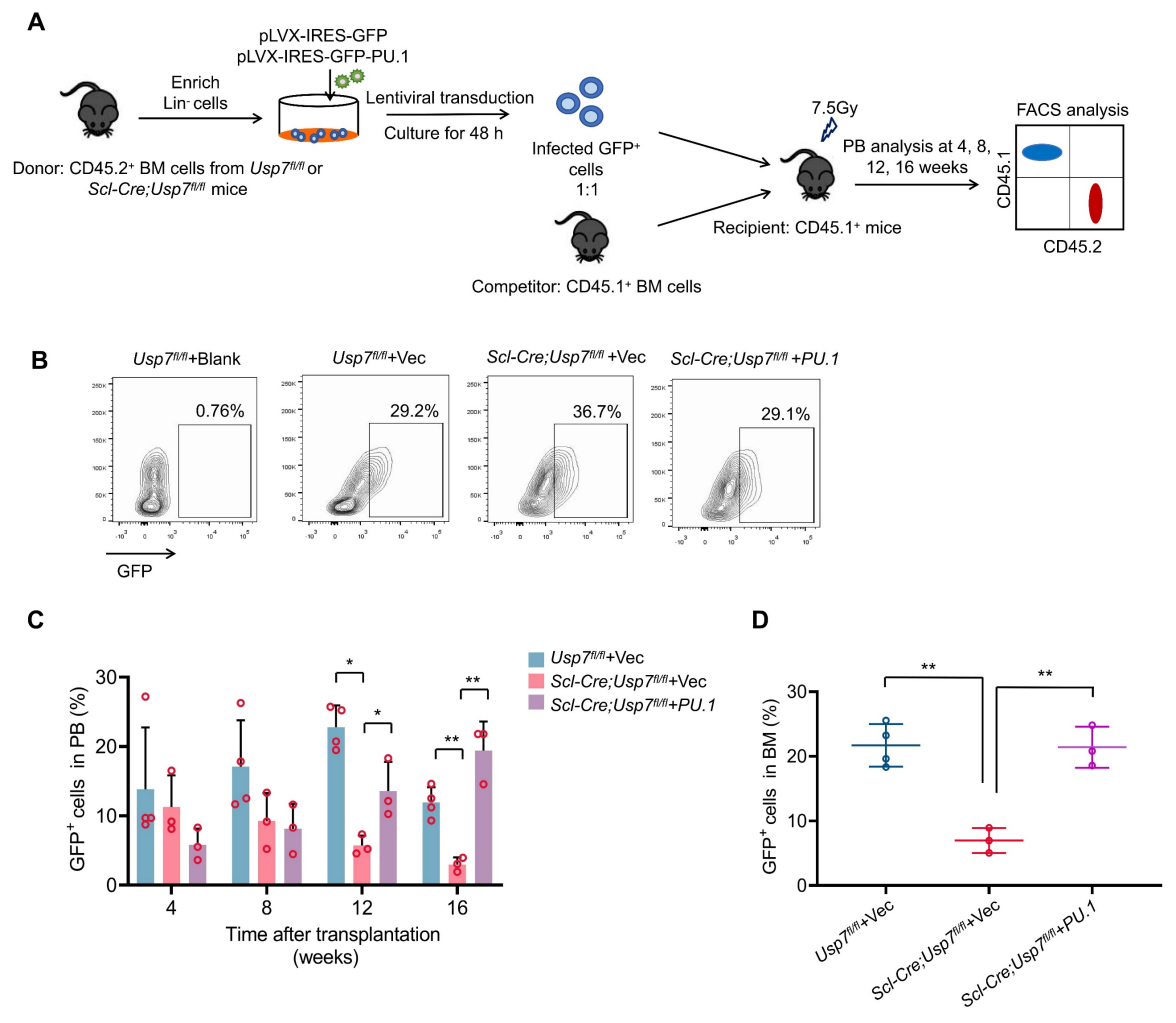
***Usp7* regulates HSC function partially by targeting PU.1.** (**A**) Schematic of the experimental design. 1 × 10^5^ lentiviruses infected control or* Scl-Cre*; *Usp7^fl/fl^* mice Lin^-^ BM donor cells were mixed with 2 × 10^5^ BM competitor cells prior to transplanted into lethally irradiated recipients. The GFP^+^ donor-derived cells in PB were assessed at different time points up to 16 weeks after transplantation. (**B**) The transduction efficiency of the virus. (**C**) Percentage of GFP^+^ donor-derived cells in PB were measured at different time points after transplant (n = 3-4). (**D**) Percentage of GFP^+^ donor-derived cells in BM were analyzed at 16 weeks after transplant (n = 3-4).

Next, we tested whether ectopic expression of PU.1 could rescue the phenotypic changes in HSCs caused by Usp7 deletion. We transduced control or *Scl-Cre*; *Usp7^fl/fl^* mice Lin^-^ BM donor cells (CD45.2^+^) with PU.1-overexpressing or control lentiviruses, then the infected donor cells were mixed with competitor cells (CD45.1^+^) prior to transplanted into lethally irradiated recipients (CD45.1^+^). Donor-derived cell reconstitution in PB was monitored at various time points for up to 16 weeks post-transplantation (Fig. 7A). The transduction efficiency of the lentiviruses was detected (Fig. 7B). Our results indicate that ectopic expression of PU.1 partially restored the repopulation capacity of *Usp7*-deficient HSPCs, as demonstrated by elevated donor-derived cell proportions in both PB and BM compared to controls (Fig. 7C-D). These data indicate that PU.1 contributes, as least partially, to the function of USP7 in HSCs.

## Discussion

USP7, a well-characterized member of the DUB family, has emerged as a critical regulator of diverse cellular processes. Previous studies have demonstrated its involvement in osteoblast/adipocyte differentiation, terminal erythroid maturation, and megakaryocytic differentiation 5, 21, 22. However, USP7's role in HSC self-renewal and hematopoiesis remains poorly understood. To address this gap, we developed *Usp7* conditional knockout mouse models to investigate its function in HSC regulation. Our findings reveal that *Usp7* deficiency results in cytopenia and impairs HSC repopulation capacity and multi-lineage differentiation. Mechanistically, we identified the transcription factor PU.1 as a novel key target of USP7-mediated regulation in HSCs. These results establish deubiquitination as a novel regulatory mechanism governing PU.1-dependent transcriptional programs in HSCs.

PU.1, a member of the ETS transcription factor family, is a well-established master regulator of hematopoietic development, primarily governing myeloid differentiation 23. Notably, hematopoietic-specific deletion of *Pu.1* leads to the absolute loss of CMPs and CLPs, while its function in maintaining HSC self-renewal capacity has been further demonstrated 24. *Pu*.*1* deficiency in HSCs impaired HSC self-renewal and long-term repopulation capacity, which could be rescued by PU.1 overexpression 25. Emerging evidence underscores that PU.1 maintains HSC quiescence during inflammatory stress by limiting protein production and suppressing cell cycle progression, thus preventing excessive HSC proliferation 20. Our study identified PU.1 as a critical USP7 target governing HSC self-renewal and differentiation for the reason that we observed several overlapping hematopoietic phenotypes between *Usp7*^-/-^ mouse models and reported *Pu.1*^-/-^ mouse models, including loss of quiescence, myeloid-biased differentiation, and repopulation capacity failure. These results implicate that the observed defects are primarily attributable to PU.1 destabilization. The partial rescue assay of *Usp7*^-/-^ HSCs defects by PU.1 overexpression further underscores PU.1's pivotal role. However, USP7's regulatory network extends far beyond this single transcription factor. In fact, our RNA-seq analysis reveals significant deregulation of several genes downstream of other hematopoietic transcription factors. These include IKZF1 26, EBF1 27, TCF3 28 and PAX5 29, all of which are critical for B cell maturation and development, a process which is compromised in PB in *Usp7*-deleted mice. We acknowledged that the possibility that USP7 functions in HSCs by stabilizing additional key transcription factors beyond PU.1. In addition, several well-characterized USP7 substrates, including p53 30, PHF8 31, and PLK1 32, are known to be key regulators of cell cycle progression and cellular proliferation—processes fundamental to HSC maintenance. These substrates exhibit temporal and context-dependent interactions with USP7. During homeostasis, USP7 may preferentially stabilize PU.1 to maintain HSC quiescence, while under proliferative stress, its activity toward cell cycle regulators like p53 could become predominant. This substrate selectivity likely explains why USP7 deletion produces more severe hematopoietic defects than PU.1 deficiency alone, as observed in our study. Future studies should address how USP7's enzymatic activity is selectively directed toward specific substrates in HSCs, and whether pharmacological USP7 inhibition can be tuned to achieve desired therapeutic effects without disrupting essential hematopoietic functions. This substrate complexity positions USP7 as a critical integrator in HSC regulation, but also presents challenges for targeted manipulation of its activity.

The finding that USP7 stabilizes PU.1 via MATH/UBL domain-dependent deubiquitination, enabling PU.1 to enforce quiescence through transcriptional activation of *Cdk1* and *E2f1* and repression of cell cycle drivers like *Myc* and *Gfi1*. This regulatory circuit explains the paradoxical hyperproliferation and functional decline of *Usp7^-/-^* HSCs, mirroring age-associated myeloid skewing linked to declining PU.1 levels. Notably, the requirement for USP7 is stage-specific—which is most pronounced in LT-HSCs—suggesting that these most primitive HSCs uniquely rely on USP7-mediated proteostasis to balance self-renewal and differentiation.

Targeting the USP7-PU.1 axis holds dual therapeutic potential. In preleukemic states (e.g., MDS), where PU.1 downregulation permits clonal expansion 33-35, our discovery that USP7 is a critical stabilizer of PU.1 suggests that targeted activation of the USP7-PU.1 axis could represent a novel therapeutic strategy. Conversely, USP7 inhibitors—though promising for cancer—risk inducing HSC exhaustion, as evidenced by our transplantation assays. Future efforts must resolve how USP7 cooperates with niche signals to regulate PU.1 dynamics and whether its activity declines with age, contributing to hematopoietic aging. In addition, key unanswered questions include: (i) how MATH/UBL domains selectively engage PU.1 amid other USP7 substrates; (ii) whether stress (e.g., inflammation, chemotherapy) modulates USP7-PU.1 interactions; and (iii) if PU.1 stabilization by USP7 reinforces its chromatin occupancy at quiescence-associated enhancers.

In summary, USP7 acts as a rheostat for HSC fate decisions by coupling protein stabilization to transcriptional control of quiescence. Our findings position USP7 inhibition as a double-edged sword in oncology and nominate PU.1 stabilization as a strategy to counteract stem cell exhaustion in aging and hematologic disorders. Deciphering how USP7 integrates with niche-derived signals will be essential for translating these insights into therapies that recalibrate HSC function.

## Supplementary Material

Supplementary figures and tables.

## Abbreviations

USP7: ubiquitin-specific protease 7
HSC: hematopoietic stem cell
DUB: deubiquitinating enzyme
LT-HSC: long-term hematopoietic stem cell
ST-HSC: short-term hematopoietic stem cell
MPP: multipotent progenitor
CMP: common myeloid progenitor
GMP: granulocyte-macrophage progenitor
MEP: megakaryocyte-erythroid progenitor
CLP: common lymphoid progenitor
LSK: Lin⁻Sca-1⁺c-Kit⁺
LK: Lin⁻Sca-1⁻c-Kit⁺
CFU-M: colony-forming unit megakaryocyte
CFU-GEMM: colony-forming unit granulocyte, erythrocyte, macrophage, megakaryocyte
BFU-E: burst-forming unit erythroid
CFU-G: colony-forming unit granulocyte
CFU-GM: colony-forming unit granulocyte-macrophage
BM: bone marrow
PB: peripheral blood
BrdU: bromodeoxyuridine
5-FU: 5-fluorouracil
MDS: myelodysplastic syndromes
Co-IP: co-immunoprecipitation
CHX: cycloheximide
