# Supplementary material for: USP7 sustains hematopoietic stem cell homeostasis partially via PU.1 stabilization
Source: Int J Biol Sci. 2026 Jan 1;22(2):1036–52. doi: 10.7150/ijbs.123712 (PMC12781850; doi:10.7150/ijbs.123712)
Supplement: Supplementary file 1 — Supplementary figures and tables. [file ijbsv22p1036s1.pdf]

Supplementary Materials

USP7 functions as a deubiquitination hub essential for hematopoietic stem cell maintenance, centered on PU.1 stabilization

Shan et al

Supplementary Tables

Supplementary Table 1. Antibodies information used for FACs.

Antigen/Kit/Dye	Company name	Catalogue number
Biotin Mouse Lineage Depletion Cocktail	BD	Cat# 51-9000794
Anti-mouse-CD3e-APC	Miltenyi Biotec	Cat#130-102-793
Anti-mouse-CD3e-PE	eBioscience	Cat# 12-0031-82
Anti-mouse-CD4-FITC	Miltenyi Biotec	Cat#130-102-779
Anti-mouse-CD8a-PE	Miltenyi Biotec	Cat#130-102-807
Anti-mouse-CD11b (Mac1)-PE	Invitrogen	Cat# RM2804
Anti-mouse-Ly-6G/-Ly-6C (Gr1)-PerCP-Cy5.5	BioLegend	Cat# 108428
Anti-mouse-CD45R/B220-APC	BioLegend	Cat# 103212
Anti-mouse-CD45.1-APC-Cy7	BioLegend	Cat# 110716
Anti-mouse-CD45.2-BV510	BioLegend	Cat# 109838
Anti-mouse-Ter119-APC	eBioscience	Cat# 17-5921-82
Anti-mouse-CD41a-PE	eBioscience	Cat# 12-0411-82
Anti-mouse-CD117 (c-Kit)-APC	eBioscience	Cat# 17-1171-82
Anti-mouse-Ly-6A/E (Sca-1)-PE-Cy7	eBioscience	Cat# 25-5981-82
Anti-mouse-Streptavidin-eFluor™ 450	eBioscience	Cat# 48-4317-82
Anti-mouse-Streptavidin-APC-Cy7	BioLegend	Cat# 405208
Anti-mouse-Streptavidin-PerCP-Cy5.5	eBioscience	Cat# 45-4317-82
Anti-mouse-CD34-PE	BioLegend	Cat# 152203

Anti-mouse-CD34-eFluor450	eBioscience	Cat# 48-0341-82
Anti-mouse-CD127-FITC	eBioscience	Cat# 11-1271-82
Anti-mouse-CD16/CD32-FITC	eBioscience	Cat# 11-0161-82
Anti-mouse-CD135 (Flt3)-PerCP-eFluor™ 710	eBioscience	Cat# 46-1351-82
Anti-mouse-CD135 (Flt3)-PE	eBioscience	Cat# 12-1351-82
Anti-mouse-Ki-67-FITC	Miltenyi Biotec	Cat#130-117-803
Annexin V-FITC Apoptosis Detection Kit I	BD	Cat# 556547
Anti-mouse-BrdU-FITC	BioLegend	Cat# 364103
CFSE	Invitrogen	Cat# C1157
Hoechst 33342	Invitrogen	Cat# H1399

Supplementary Table 2. List of primers for mice genotyping and gene deletion efficiency.

Genotyping primers	Sequence
Usp7$^{fl/fl}$-F/*Usp7*^{KO}-F	CCTTGGTTTCCTGTGCATTTTGTC
Usp7$^{fl/fl}$-R	AAGAGGGTACCATTCACCCATCAC
Mx1-Cre-F	CAGCATTGCTGTCACTTGGTC
Mx1-Cre-R	ATTTGCCTGCATTACCGGTCG
Scl-Cre-ER-F	GAACCTGAAGATGTTCGCGAT
Scl-Cre-ER-R	ACCGTCAGTACGTGAGATATC
Usp7^{KO}-R	CACAATGTTAGGCTCCTTGGTACAG

Supplementary Table 3. List of primers used in qRT-PCR analysis.

Gene	Primer	Sequence (5′-3′)
mouse *Usp7*	Forward	5′-TCCTCAGCAGTTGGTGGAACGA-3′
	Reverse	5′-GCCACAAAACTGGTCCTCTGCA-3′

mouse *Pu.1*	Forward	5′-ATGTTACAGGCGTGCAAAATGG-3′
	Reverse	5′-TGATCGCTATGGCTTTCTCCA-3′
mouse *Gapdh*	Forward	5′-CTTAGCCCCCCTGGCCAAG-3′
	Reverse	5′-TGGTCATGAGCCCTTCCACA-3′
mouse *Myc*	Forward	5′-GCTGTTTGAAGGCTGGATTTC-3′
	Reverse	5′-GATGAAATAGGGCTGTACGGAG-3′
mouse *Gfi1*	Forward	5′-TGGCTAAACTAAGCCACGCA-3′
	Reverse	5′-CTCCCCTTCCCTACCAAAGC-3′
mouse *Cdk1*	Forward	5′-GACAGAGAGGGTCCGTCGTA-3′
	Reverse	5′-TGGCCAGTGACTCTGTGTCT-3′
mouse *E2f1*	Forward	5′-CGATTCTGACGTGCTGCTC-3′
	Reverse	5′-CAGCGAGGTACTGATGGTCA-3′
mouse *Flt3*	Forward	5′-ATCCTTCCCCAACCTGACTT-3′
	Reverse	5′-TTGCCACCCATGTTCTGATA-3′
mouse *Gata1*	Forward	5′-GAAGCGAATGATTGTCAGCA-3′
	Reverse	5′-TTCCTCGTCTGGATTCCATC-3′
mouse *c-Kit*	Forward	5′-GACGCAACTTCCTTATGATC-3′
	Reverse	5′-TGGTTTGAGCATCTTCACGG-3′

Supplementary Figures and Figure legends

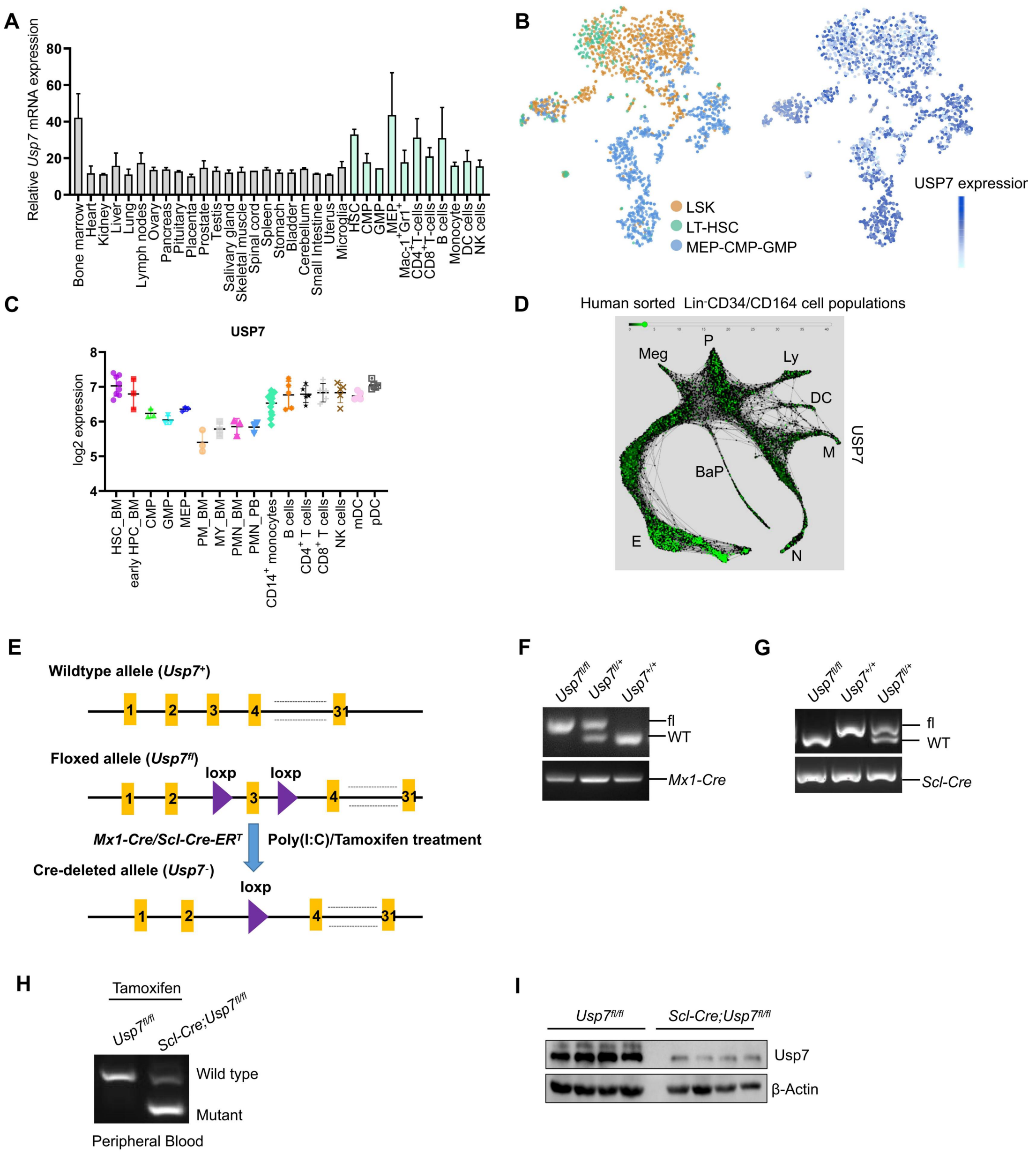

Figure S1. USP7 was expressed at high levels in hematopoietic stem and progenitor cells (HSPCs). (**A**) Analysis of *Usp7* expression levels in various tissues, organs, and cell subpopulations of mice using the BioGPS public gene information database (Dataset: GeneAtlas MOE430, gcrma; Probeset: 1454949_at). Data were normalized. (**B**) Analysis of *Usp7* expression levels in the scRNA-seq dataset of

mouse hematopoietic stem/progenitor cells using the Single Cell Expression Atlas database. Each dot on the graph represents a single cell, with different colors indicating different cell types. (**C**) The BloodSpot database (Dataset: Normal human hematopoiesis (HemaExplorer)) provides the expression profile of USP7 in various cell subpopulations of the normal human hematopoietic system based on gene microarray data. The horizontal line represents the average expression value for that cell type. (**D**) Analysis of USP7 expression levels in the scRNA-seq dataset of human Lin^-CD34/CD164 cells. The figure shows the SPRING plot of the single-cell transcriptome of human Lin^-CD34/CD164 cells, where each dot represents a single cell, and the labels at the edges of the trajectory indicate the transcriptional stages of early lineage commitment. (**E**) Schematic of the *Usp7* floxed allele showing the deletion of floxed exon 3 following Cre recombinase activity. Use of *Mx1-Cre* results in specific deletion in HSCs following poly (I:C) treatment. Use of *Scl-Cre*-ER^T results in specific deletion in HSCs following tamoxifen treatment. (**F**) Representative genotyping results of WT, heterozygote, and floxed *Usp7* mice, with *Mx1-Cre* recombinase. (**G**) Representative genotyping results of WT, heterozygote, and floxed *Usp7* mice, with *Scl-Cre* recombinase. (**H**) Representative genotyping results confirming *Usp7* deletion in PB cells from control and *Scl-Cre; Usp7*$^{fl/fl}$ mice one week after the last dose of tamoxifen. (**I**) *Usp7* deletion was examined by western blotting in total BM cells from control and *Scl-Cre; Usp7*$^{fl/fl}$ mice (n = 4).

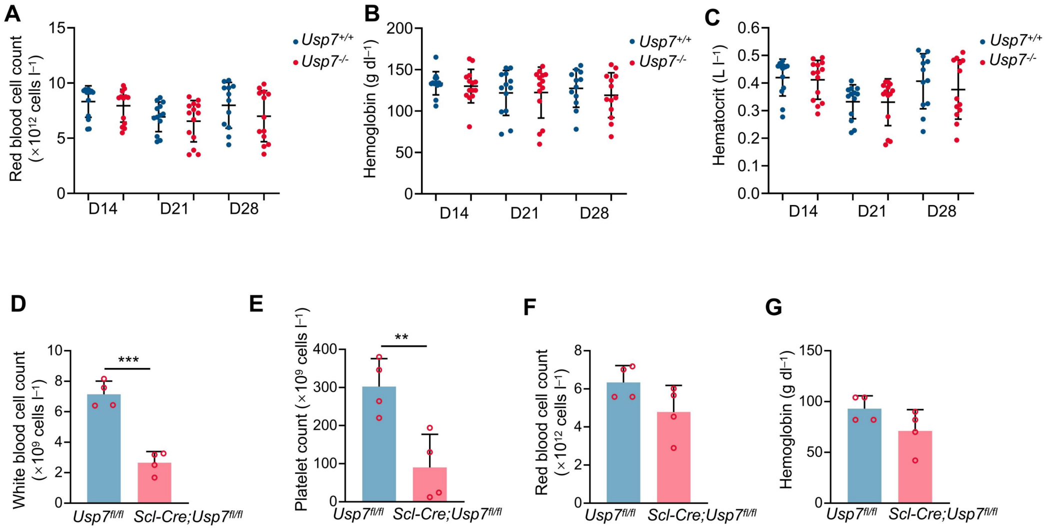

Figure S2. ***Usp7*** **deficiency reduces the whole blood counts and platelet counts but does not affect red blood cell parameters. (A-C)** Whole blood counts of red blood cell count (**A**), hemoglobin (**B**), and hematocrit (**C**) counts from *Usp7*$^{+/+}$ and *Usp7*$^{-/-}$ mice at indicated times after the last dose of poly (I:C) (n = 13-14). (**D-G**) Whole blood counts of white blood cell (**D**), platelet (**E**), red blood cell count (**F**) and hemoglobin (**G**) counts from control and *Scl-Cre; Usp7*$^{fl/fl}$ mice at one week after the last dose of tamoxifen (n = 4).

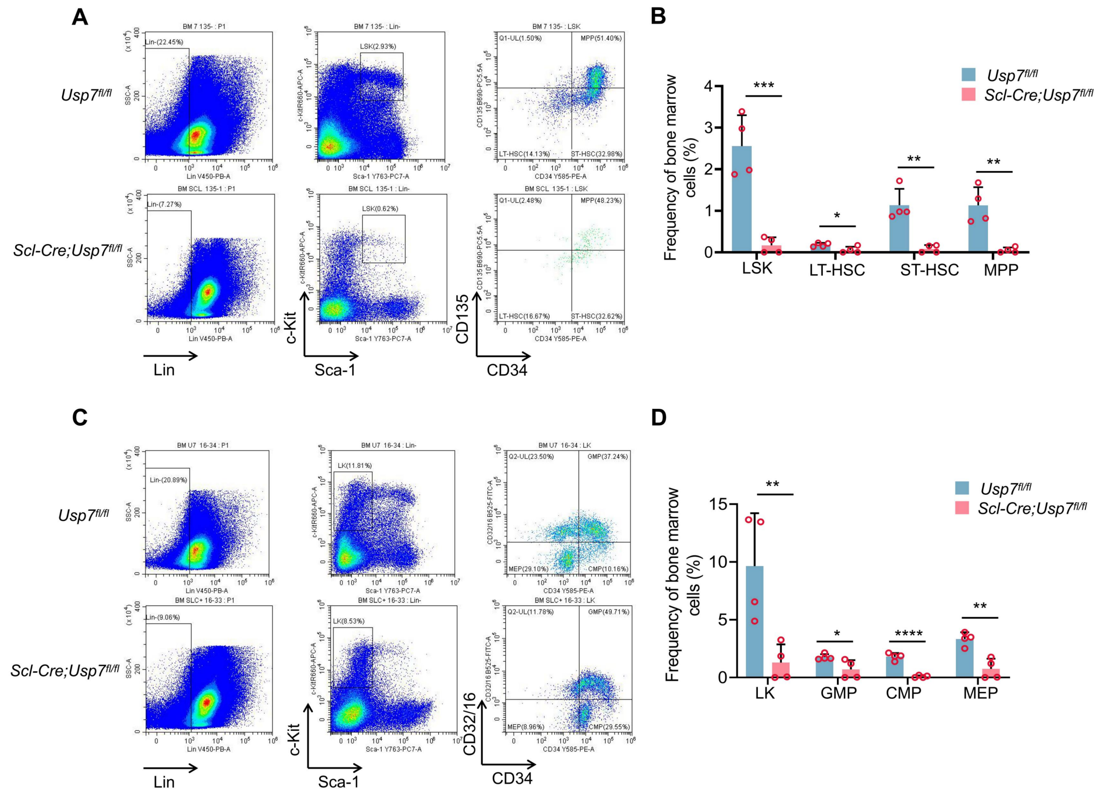

Figure S3. ***Usp7*** **deficiency impairs the HSCs numbers using** ***Scl-Cre*****;** ***Usp7***$^{fl/fl}$ **mouse model.** (**A-B**) Representative FACS plots (**A**) and frequencies of LSK cells (**B**), LT-HSCs, ST-HSCs, and MPPs (**B**) in BM from control and *Scl-Cre*; *Usp7*$^{fl/fl}$ mice at one week after the last tamoxifen injection (n = 4). (**C-D**) Representative FACS plots (**C**) and frequencies of LK cells (**D**), CMPs , GMPs, and MEPs (**D**) in BM from control and *Scl-Cre*; *Usp7*$^{fl/fl}$ mice at one week after the last tamoxifen injection (n = 4).

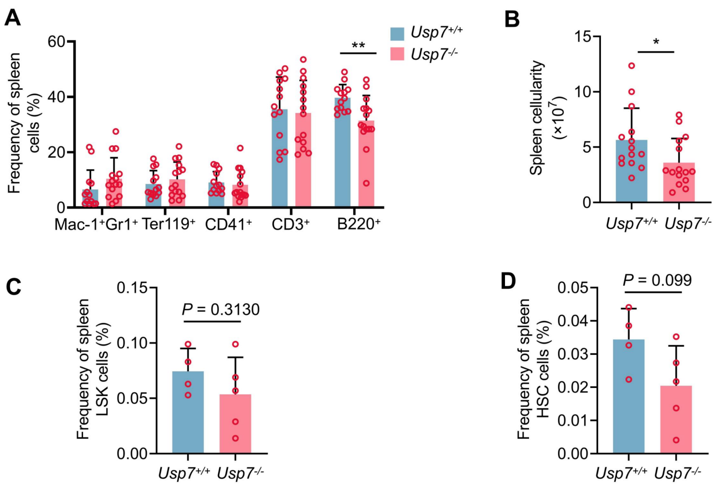

Figure S4. *Usp7* deficiency does not affect hematopoiesis in the spleen. (A) Frequency of differentiated cells in the spleen from *Usp7*$^{+/+}$ and *Usp7*$^{-/-}$ mice at 4 weeks after poly (I:C) injection (n = 13-15). (**B**) Total numbers of spleen cells were counted in *Usp7*$^{+/+}$ and *Usp7*$^{-/-}$ mice at 4 weeks after poly (I:C) injection (n = 14-15). (**C**) Frequency of LSK cells in spleen from *Usp7*$^{+/+}$ and *Usp7*$^{-/-}$ mice at 4 weeks after poly (I:C) injection (n = 4-5) . (**D**) Frequency of LT-HSCs in spleen from *Usp7*$^{+/+}$ and *Usp7*$^{-/-}$ mice at 4 weeks after poly (I:C) injection (n = 4-5).

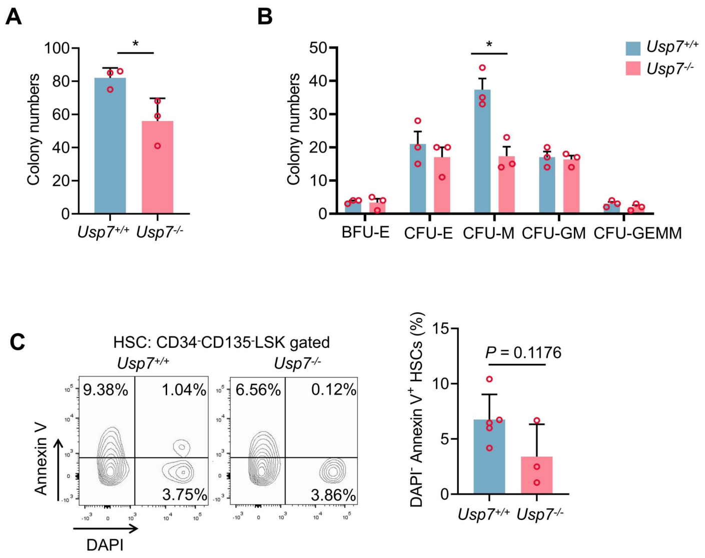

Figure S5. *Usp7* deficiency reduces the colony formation ability of BM cells, but does not affect the apoptotic states of HSCs. (**A**) The colony forming abilities were measured with total BM cells from *Usp7*$^{+/+}$ or *Usp7*$^{-/-}$ mice. 2×10^4 cells were seeded in the 6-well plate and the colony numbers in each well were evaluated after culturing for 10 days (n = 3). (**B**) The colony-forming units (CFUs) were scored as granulocyte, erythrocyte, monocyte and megakaryocyte (GEMM), macrophage (GM), megakaryocyte (M), granulocyte (G) or erythrocyte (E) colonies (n = 3). (**C**) Cell apoptosis of LT-HSCs in BM from *Usp7*$^{+/+}$ and *Usp7*$^{-/-}$ mice at 4 weeks after poly (I:C) injection (n = 3-5) . Representative FACS plots for double Annexin V/DAPI stainings (left) and percentages of Annexin V^{+}/DAPI^{-} cells were analyzed.

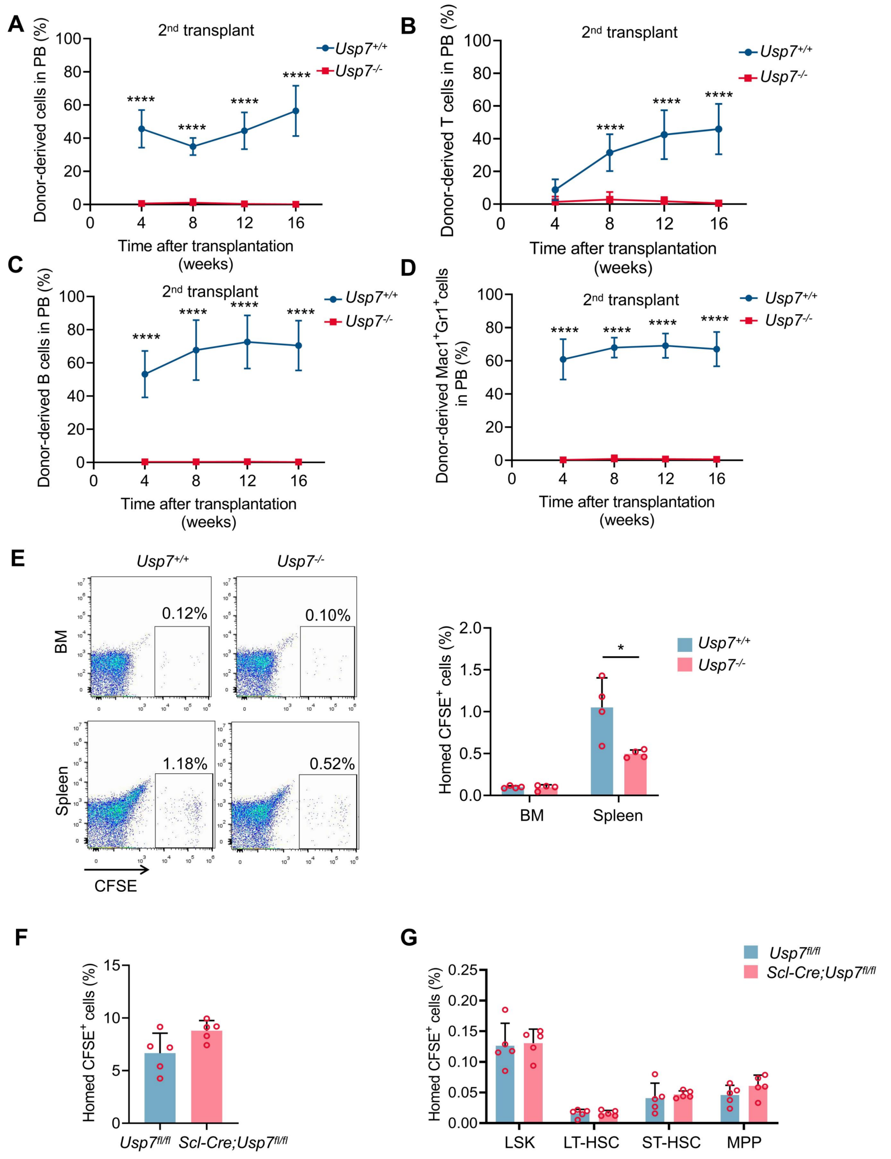

Figure S6. Loss of *Usp7* disrupts the long-term repopulation capacity of HSCs, and does not influence the homing of HSCs. (A-D) Percentage of donor-derived cells (**A**), T cells (**B**), B cells (**C**), and Mac-1$^+$Gr-1$^+$ myeloid cells (**D**) in PB of recipient mice were measured at different time points in a serial competitive BM transplantation (n = 7-9). (**E**) 1× 10^7 BM cells from *Usp7*$^{+/+}$ and *Usp7*$^{-/-}$ mice were labeled with CFSE and transplanted into lethally irradiated recipient mice. Percentage of donor cells in the recipients was detected 18 h post-transplantation. Representative FACS plots for homing (left) and percentages of CFSE$^+$ cells (right) in the BM and

spleen were showed (n = 4). (**F-G**) 2×10^7 BM cells from control and *Scl-Cre*; *Usp7$^{fl/fl}$* mice were labeled with CFSE and transplanted into lethally irradiated recipient mice. Percentages of $CFSE^+$ cells in BM (**F**) as well as $CFSE^+$LSK cells, $CFSE^+$LT-HSCs, $CFSE^+$ST-HSCs, $CFSE^+$MPPs in BM (**G**) were analyzed at 18h post-transplantation (n = 5).

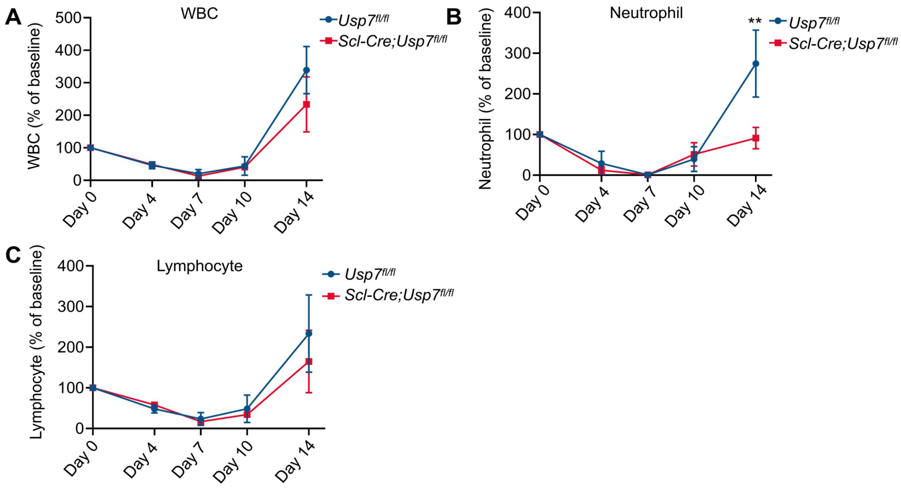

Figure S7. *Usp7* deficiency exhibited faster recovery capability in the 5-FU killing assay. (A-C) The control or *Scl-Cre*;*Usp7*$^{fl/fl}$ mice were intraperitoneally injected with a single injection of 5-FU at a dose of 150 mg/kg. PB samples were collected from the mice at different time points for analysis of white blood cells (**A**), neutrophils (**B**) and lymphocytes (**C**) .

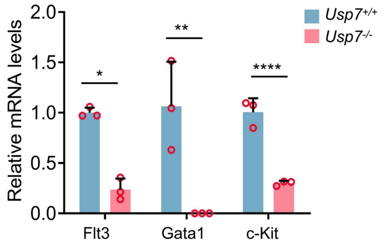

Figure S8. ***Usp7*** **deficiency downregulated the target genes from the hematopoietic cell lineage pathway.** qPCR analysis of the indicated genes in *Usp7*$^{+/+}$ or *Usp7*$^{-/-}$ HSCs (n = 3).
